# A transcribed enhancer dictates mesendoderm specification in pluripotency

**DOI:** 10.1038/s41467-017-01804-w

**Published:** 2017-11-27

**Authors:** Michael Alexanian, Daniel Maric, Stephen P. Jenkinson, Marco Mina, Clayton E. Friedman, Ching-Chia Ting, Rudi Micheletti, Isabelle Plaisance, Mohamed Nemir, Damien Maison, Jasmin Kernen, Iole Pezzuto, Dominic Villeneuve, Frédéric Burdet, Mark Ibberson, Stephen L. Leib, Nathan J. Palpant, Nouria Hernandez, Samir Ounzain, Thierry Pedrazzini

**Affiliations:** 1 Experimental Cardiology Unit, Department of Cardiovascular Medicine, University of Lausanne Medical School, 1011 Lausanne, Switzerland; 2 Neuroinfectiology Laboratory, Institute for Infectious Diseases, University of Bern, 3001 Bern, Switzerland; 3 Department of Computational Biology, University of Lausanne, 1011 Lausanne, Switzerland; 4 Swiss Institute of Bioinformatics, 1015 Lausanne, Switzerland; 5 The Institute for Molecular Bioscience, University of Queensland, 4067 Brisbane, Australia; 6 Center for Integrative Genomics, Faculty of Biology and Medicine, University of Lausanne, 1015 Lausanne, Switzerland

## Abstract

Enhancers and long noncoding RNAs (lncRNAs) are key determinants of lineage specification during development. Here, we evaluate remodeling of the enhancer landscape and modulation of the lncRNA transcriptome during mesendoderm specification. We sort mesendodermal progenitors from differentiating embryonic stem cells (ESCs) according to *Eomes* expression, and find that enhancer usage is coordinated with mesendoderm-specific expression of key lineage-determining transcription factors. Many of these enhancers are associated with the expression of lncRNAs. Examination of ESC-specific enhancers interacting in three-dimensional space with mesendoderm-specifying transcription factor loci identifies MesEndoderm Transcriptional Enhancer Organizing Region (*Meteor*). Genetic and epigenetic manipulation of the *Meteor* enhancer reveal its indispensable role during mesendoderm specification and subsequent cardiogenic differentiation via transcription-independent and -dependent mechanisms. Interestingly, *Meteor*-deleted ESCs are epigenetically redirected towards neuroectodermal lineages. Loci, topologically associating a transcribed enhancer and its cognate protein coding gene, appear to represent therefore a class of genomic elements controlling developmental competence in pluripotency.

## Abstract (web summary)

Long noncoding RNAs (lncRNAs) are key regulators of lineage specification during development. Here, the authors investigate remodeling of enhancers and regulation of the lncRNA transcriptome during mesendoderm specification, and identify a pluripotent stage-specific transcribed enhancer controlling adoption of the mesendodermal cell fate.

## Introduction

A complex molecular program controls cardiac differentiation and morphogenesis during development^1^. Understanding the gene regulatory networks (GRNs) governing cardiac organogenesis could lead to innovations for the treatment of both inherited and acquired heart disease^1,2^. In addition, induction of cardiac regeneration via either cell-based therapies or reactivation of dormant endogenous mechanisms has engendered considerable interest over the past decade^3^. Both approaches require a deep knowledge of the molecular and cellular events regulating cardiac specification and differentiation. However, transcriptional profiling of individual transient cellular intermediates in the developing heart is extremely challenging and requires the use of in vitro cellular systems^4^. During gastrulation, epiblast cells undergo epithelial-to-mesenchymal transition (EMT) and ingress through the primitive streak (PS)^5^. This transient precursor cell population, referred to as the mesendoderm (ME), is the source of the definitive endoderm and the mesoderm. Nascent mesoderm cells then rapidly migrate from the posterior side of the embryo to the anterior side, become specified to the cardiac lineage, and ultimately generate the embryonic heart^6^. Mesendodermal precursors are characterized by the expression of genes such as Eomesodermin (*Eomes*), Goosecoid (*Gsc*), and LIM-homeobox1 (*Lxh1*)^7^. In particular, *Eomes*, a T-box transcription factor (TF), is critical for ME specification between embryonic day 6.5–7.5 in the mouse. *Eomes* expression marks the earliest cardiac mesoderm and dictates the formation of cardiac precursors through regulating the master TF Mesoderm posterior 1 (*Mesp1*)^8^. The transient formation of the ME is under the control of a specialized GRN consisting of cell-fate determining TFs that interact at target sequences known as enhancers^7^. Moreover, enhancers are an important class of distal regulatory elements that are key information processing units within the genome, controlling the precise spatiotemporal expression of their target protein-coding genes (PCGs)^9^. Recently, regions of the mammalian genome comprising multiple enhancers have been identified and termed super-enhancers (SEs)^10,11^. SEs are typically an order of magnitude larger than typical-enhancers (TEs) and are highly enriched with regulatory TFs and chromatin marks (i.e., H3K27ac). They are master regulators of developmental and cell identity genes, which are critical for cell fate determination and differentiation^10^. Through integrating upstream context-specific developmental signals, TF-bound TEs and SEs mediate appropriate gene programs required to mark and specify mesendodermal fate^7,9,11^.

Progress in high-throughput sequencing has advanced our understanding of genome organization and regulation. Only 2% of the genome appears to code for proteins. The remaining 98% represents the noncoding fraction of the genome. Most of the noncoding genome is transcribed into RNAs^12^. In particular, long noncoding RNAs (lncRNAs) represent an important class of regulatory molecules. LncRNAs are typically transcribed by RNA polymerase II and are usually multiexonic and polyadenylated^13^. Interestingly, lncRNAs have been shown to be expressed in unique cell types, for instance across various stages of differentiation, suggesting their involvement in regulating cell fate^13^. An important subset of RNAs is associated with enhancers, and named enhancer RNAs (eRNAs)^14,15^. They exist as two different transcripts: bidirectional non-polyadenylated transcripts; and unidirectional, multiexonic, spliced, polyadenylated transcripts. Recent studies have demonstrated that targeted degradation of eRNAs is sufficient to reduce expression of adjacent PCGs^14^. In particular, eRNAs appear to be involved in the formation and stabilization of the loop between the enhancer and the promoter in a typical *Cis*-regulatory manner^14^. In addition, chromatin conformation capture approaches have shown that the genome is hierarchically organized into larger domains known as topologically associating domains (TADs)^16^. TADs are linear DNA segments that form independent units in nuclear three-dimensional space. Importantly, TAD boundaries are well conserved across species and cell-types. Disrupting boundaries results in spurious interactions between promoters and enhancers normally residing in different TADs, leading to transcriptional dysregulation^17^. Interestingly, transcribed DNA elements, including enhancers, are emerging as potential regulators of TAD formation^18^. However, an important debate revolves around the nature of lncRNA functions. Whether the simple act of transcription from the lncRNA locus or the mature lncRNA transcript results in observed phenotypes is still unclear^19^.

Within this context, mouse embryonic stem cells (mESCs) harboring reporter genes transcribed from developmental TF promoters represent a unique system to model the formation of cellular intermediates such as the ME precursors. More precisely, the enhancer and the lncRNA landscapes governing ME specification, and thereby cardiac differentiation has not been investigated. Here, we use an *Eomes* reporter mESC line to assess remodeling of the enhancer landscape and to profile the lncRNA transcriptome during ME specification^20^. We identify a large number of previously uncharacterized enhancer-associated lncRNAs. Examination of ESC-specific enhancer-associated lncRNA loci within mesendodermal TADs identified an *Eomes*-interacting locus, which was named MesEndoderm Transcriptional Enhancer Organizing Region (*Meteor*). Interestingly, the lncRNA associated to the *Meteor* enhancer corresponds to a previously described pluripotency associated lncRNA^21,22^. *Meteor* deletion and epigenetic manipulation reveals its indispensable role during ME determination and subsequent cardiogenic differentiation, supporting a predetermined role for this class of genomic elements in programming developmental competence and ESC specification during development.

## Results

### Early cell fate specification in mesendodermal progenitors

We utilized an *Eomes* reporter mESC line engineered to carry an EGFP cassette inserted into the transcriptional start site of the endogenous *Eomes* gene (*Eomes* ^EGFP^ ESCs) (Supplementary Fig. 1a)^20^. These cells were induced to differentiate using the hanging drop model^23^. This method allows the stepwise differentiation of ESCs towards the cardiogenic lineage, generating mesendoderm precursor cells (MEPC), cardiac precursor cells (CPCs) and ultimately differentiated cardiomyocytes (CMs) (Supplementary Fig. 1b). Initially, embryoid bodies (EBs) were harvested every 12 h during a 10-day period. Differentiation was accompanied by the downregulation of pluripotency-associated genes, the transient induction of ME and cardiac mesoderm specifying TFs *Eomes*, *T* and *Mesp1*, and finally the expression of markers of cardiac differentiation including *Myh6* and *Myh7* (Supplementary Fig. 1c). Terminal differentiation resulted in a significant number of beating EBs at both day 8 and 10 (Supplementary Fig. 1d). Importantly, the *Eomes* ^EGFP^ reporter was able to mark *Eomes*-expressing cells at day 3 of differentiation, a time point at which *Eomes* is maximally expressed and specifies the nascent mesoderm (Supplementary Fig. 1e). Flow cytometry analysis indicated that half of the differentiating cells at day 3 commit to ME (Supplementary Fig. 1f, g).

We next isolated *Eomes*-positive (*Eo* ^*+*^) and *Eomes*-negative (*Eo* ^*−*^) cells at day 3 using fluorescence-activated cell sorting (FACS) (Supplementary Fig. 2a) and measured the expression of pluripotency genes (*Nanog*, *Sox2, Oct4*), markers of ME (*Eomes*, *Lhx1*, *Mixl1*), cardiac mesoderm (*Mesp1*, *Gata4*, *Nkx2-5*) and neuroectoderm (*Pax6, Nkx6-3*, *Neurog3*) (Supplementary Fig. 2b). As compared to undifferentiated ESCs, significant expression of mesendodermal and cardiac mesodermal genes was measured in *Eo* ^*+*^ cells. In addition, neuroectoderm gene expression was higher in *Eo* ^*−*^ cells than in *Eo* ^*+*^ cells. To validate these subpopulations for subsequent genome-wide chromatin immunoprecipitation followed by sequencing (ChIP-Seq) analysis, we performed ChIP-qPCR using antibodies against H3K4me3 (associated with active promoters) and H3K27Ac (associated with active enhancers). Primers were designed within known promoter and enhancer regions associated with pluripotency (*Nanog*) and ME (*Eomes*) (Supplementary Fig. 2c)^24^. As expected, the *Nanog* promoter and the associated distal enhancer were enriched with H3K4me3 and H3K27Ac respectively in pluripotent ESCs. On the other hand, the *Eomes* promoter and enhancer were enriched with the H3K4me3 and H3K27Ac marks in the *Eo* ^*+*^ sorted cells (Supplementary Fig. 2d). Our data thus indicates that *Eo* ^*+*^ cells express a unique transcriptional and enhancer signature reflecting their potential to become ME-derived lineages, including cardiac mesoderm.

### Transcriptome assessment during mesendoderm specification

**Fig. 1:**
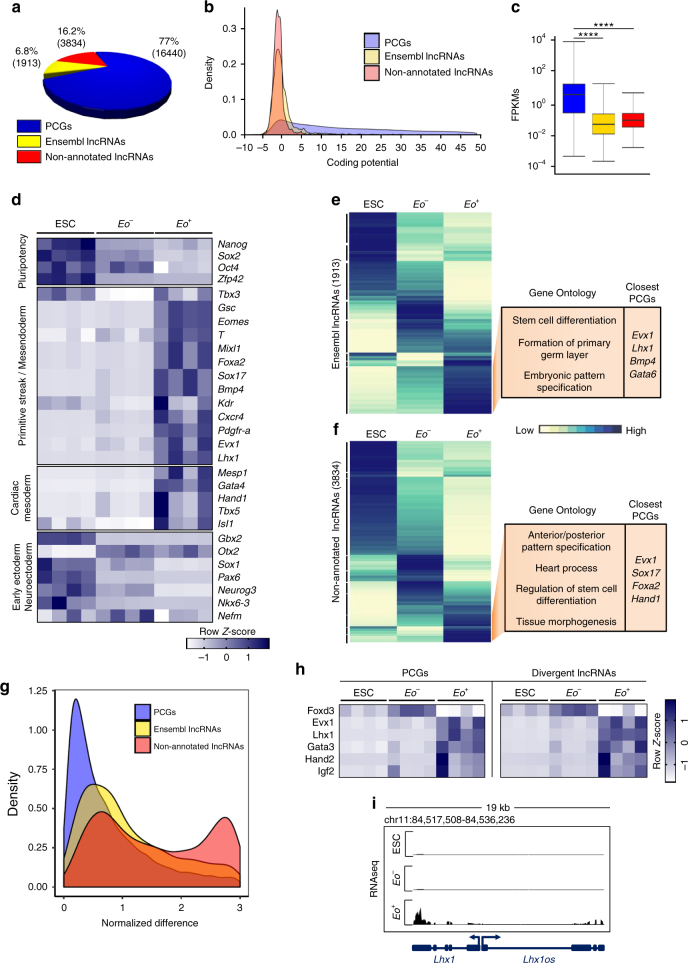
Global assessment of the transcriptome during mesendoderm specification. **a** Pie chart showing composition of the Poly (A)^+^ transcriptome, Protein Coding Genes (PCG, blue), Ensembl lncRNAs (yellow) and non-annotated lncRNAs (red). **b** Kernel density plot of coding potential (Gene ID score) of PCGs, Ensembl lncRNAs and non-annotated lncRNAs. **c** Box plot whiskers of transcript abundance (FPKM) of PCGs (blue), Ensembl lncRNAs (yellow) and non-annotated lncRNAs (red). *p* values were calculated using a two-tailed *t* test. (****P < 0.0001). **d** Expression heatmap of representative PCGs in ESC, *Eo* ^*−*^ and *Eo* ^*+*^. Markers of pluripotency, Primitive Streak/Mesendoderm (PS/ME), cardiac mesoderm and early ectoderm/neuroectoderm are shown. **e** Hierarchical clustering of Ensembl lncRNA expression across ESC, *Eo* ^*−*^ and *Eo* ^*+*^. Enriched GO terms and example PCGs are shown to the right. **f** Hierarchical clustering of non-annotated lncRNA expression across ESC, *Eo* ^*−*^, and *Eo* ^*+*^. Enriched GO terms and example PCGs are shown to the right. **g** Kernel density plot of the specificity of PCG and lncRNA assessed by quantifying the normalized difference of expression in the three conditions (ESC, *Eo* ^*−*^ *, Eo* ^*+*^). **h** Co-activation of selected PCGs and divergent lncRNAs involved in ME specification. **i** RNAseq reads in ESC, *Eo* ^*−*^ and *Eo* ^*+*^ at the *Lhx1 locus*. A divergent lncRNA (*Lhx1os*) is shown

To characterize the transcriptome, and in particular the long noncoding transcriptome, in pluripotent ESCs and in sorted *Eo* ^*+*^ and *Eo* ^*−*^ cells at day 3 of differentiation, we performed very deep sequencing (>500 million reads per sample) coupled to ab initio reconstruction (Supplementary Fig. 3a). We integrated our reconstructed transcripts with the Ensembl gene annotation. Using this pipeline, we reconstructed 22,187 transcripts of which 16,440 corresponded to annotated PCGs. In addition, 5747 lncRNAs were identified. This included 1913 previously annotated lncRNAs and 3834 multiexonic non-annotated lncRNAs (Fig. 1a; Supplementary Data 1). The non-annotated lncRNAs encode minimal and comparable protein coding potential to Ensembl-annotated lncRNAs (Fig. 1b). At the end, we disregarded any transcripts with a coding potential score greater than 4. Ensembl and non-annotated lncRNAs were globally expressed at significantly lower levels than PCGs (Fig. 1c). Unsupervised hierarchical clustering of all PCGs, Ensembl annotated lncRNAs and non-annotated lncRNAs identified three distinct clusters in ESCs, *Eo* ^*−*^ and *Eo* ^*+*^ cells (Supplementary Fig. 3b), demonstrating that the transcriptome was representative of the developmental events associated with ME specification.

LncRNA exons were determined to be less conserved than PCG exons. In contrast, promoters of both annotated and non-annotated lncRNAs were significantly more conserved than the neutrally evolving genomic background (Supplementary Fig. 3c). We proceeded to determine whether this reflected evolutionary constraint at lncRNA promoters impacting on specific developmental cellular intermediates during cardiac mesoderm differentiation. We used in this analysis the current RNA-Seq datasets and two previously generated datasets for cardiac precursor cells and the adult heart^25,26^. We first identified the proximal promoter DNA sequences for PCGs, Ensembl and non-annotated lncRNAs in each of these five populations, i.e., ESCs, *Eo* ^*−*^, *Eo* ^*+*^, CPC, and adult heart, defining four inferred branch points. We next calculated the mean level of evolutionary constraint for each set of promoters (Supplementary Fig. 3d). We found that promoters of ME-associated transcripts were significantly more constrained than those specific to populations at other stages of cardiac differentiation. Furthermore, non-annotated lncRNA promoters specific to *Eo* ^*+*^ cells were significantly more constrained than promoters of non-annotated lncRNAs expressed in *Eo* ^*−*^ cells with this difference not detectable for Ensembl lncRNAs. Interestingly, promoter conservation at non-annotated lncRNAs recapitulates the hourglass model of development^27^, and supports therefore an evolutionary conserved role for non-annotated lncRNA loci with respect to ME specification.

We next analyzed the expression of a series of PCGs that are typically associated with pluripotency, primitive streak/ME, cardiac mesoderm, early ectoderm and neuroectoderm. ME and cardiac mesoderm TFs, including for instance *Eomes*, *T*, and *Mesp1*, were highly enriched in *Eo* ^*+*^ cells while pluripotency TFs were enriched in ESCs (Fig. 1d). In contrast, the *Eo* ^*−*^ cells were not associated with a well-defined transcription signature. Importantly, PCG expression was characterized by stage-specific induction or repression of key lineage-determining TFs (Supplementary Fig. 4a). Unsupervised clustering yielded specific groups of PCGs expressed specifically within each cell population (Supplementary Fig. 4b). These cell population-specific clusters were associated to expected gene ontology (GO) terms (Supplementary Data 2). For example, PCGs enriched in *Eomes*-expressing cells were strongly associated with processes linked to gastrulation and mesoderm formation. Extending this analysis to the long noncoding transcriptome, we found that both Ensembl and non-annotated lncRNAs, encompassing all known biotypes (Supplementary Fig. 4c), exhibited striking stage-specific expression (Fig. 1e, f; Supplementary Data 3). We determined GO terms for the nearest PCGs relative to Ensembl and non-annotated lncRNAs enriched in *Eomes*-expressing cells (Fig. 1e, f). Interestingly, non-annotated lncRNAs enriched in *Eo* ^*+*^ cells were proximal to PCGs specifically linked to heart processes, implicating non-annotated lncRNAs as potentially important and specific regulators of cardiac mesoderm specification and differentiation. Furthermore, non-annotated lncRNAs exhibited greater cell type-specific expression as compared to PCGs and annotated lncRNAs (Fig. 1g). Recently, it has been demonstrated that the expression of the lncRNA biotype known as divergent lncRNAs strongly correlates with that of their cognate developmental PCGs^28,29^. We therefore selected and assessed the expression of six key ME and ectoderm TFs, in addition to their divergently expressed lncRNAs (Fig. 1h). A prototypic example is *Lhx1* and its divergent noncoding transcript *Lhx1os* (Fig. 1i). In agreement with previous findings, divergent lncRNAs were exquisitely correlated in their expression with their paired PCG (Supplementary Fig. 4d)^28,29^.

### Tissue-enrichment characteristics of mesendodermal lncRNAs

**Fig. 2:**
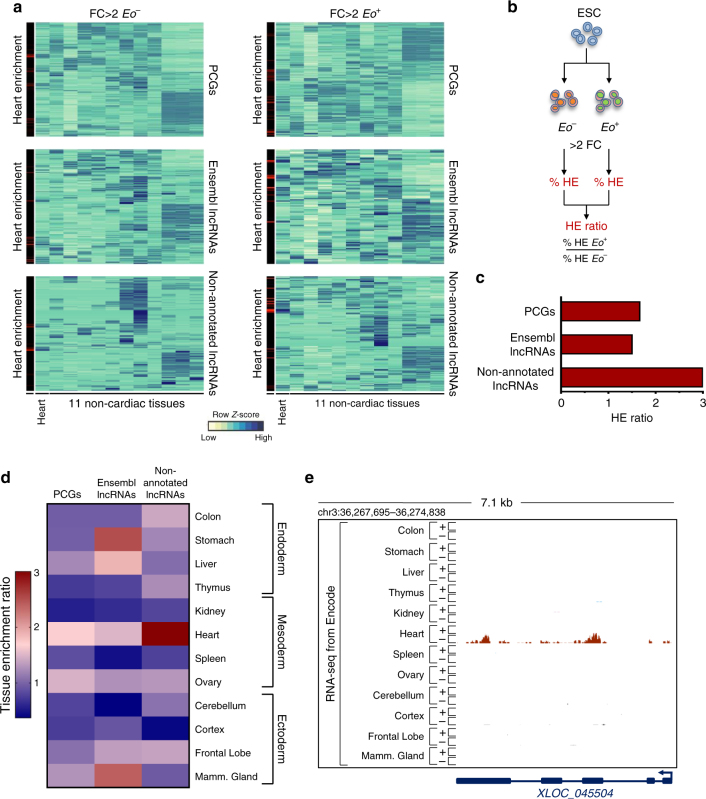
Non-annotated lncRNAs have unique tissue enrichment characteristics. **a** Clustering of *Eo* ^*−*^ and *Eo* ^*+*^ enriched PCGs, Ensembl lncRNAs and non-annotated lncRNAs across adult heart and 11 non-cardiac mouse ENCODE tissues. Left-hand panels highlight individual heart-enriched transcripts in red. **b** Schematic illustration of the pipeline utilized to calculate the Heart Enrichment (HE) score. *Eo* ^*−*^ enriched transcripts (>2FC over *Eo* ^*+*^) and *Eo* ^*+*^ enriched transcripts (>2FC over *Eo* ^*−*^) were defined. Percentage of heart enriched transcripts was defined by comparing the expression of *Eo* ^*−*^ enriched transcripts and *Eo* ^*+*^ enriched transcripts in the adult heart vs. 11 non-cardiac tissues (The ENCODE Project Consortium, 2011). HE ratio was calculated by dividing the percentage of HE transcripts in *Eo* ^+^ over the percentage of HE transcripts in *Eo* ^*−*^. **c** HE ratio in PCGs, Ensembl lncRNAs and non-annotated lncRNAs. **d** The same pipeline illustrated in (**b**) was applied to calculate tissue enrichment ratio for 12 mouse ENCODE tissues. Double gradient heatmap showing tissue enrichment ratio for the 12 mouse ENCODE tissues. **e** Strand-specific RNAseq data from 12 ENCODE tissues is shown for an example non-annotated lncRNA enriched in *Eo* ^*+*^

Very deep sequencing of RNA samples obtained from defined cell subpopulations allows the identification of noncoding transcripts that usually escape previous annotation^30^. These transcripts are more likely to demonstrate high cell-type and tissue specificity. Many of the non-annotated lncRNAs exhibited particular enrichment in ME-committed cells (Fig. 1f). We therefore suspected that these transcripts could also be enriched in ME-derived tissues, including the heart. To evaluate this possibility, we selected all transcripts that were significantly expressed in either the *Eo* ^*−*^ or *Eo* ^*+*^ cells. We then computationally mapped twelve mouse ENCODE RNA-Seq datasets^31^ obtained from the heart and eleven non-cardiac tissues using the Ensembl and our non-annotated lncRNA annotations. Expression data in Fig. 2a identify heart-enriched transcripts in *Eo* ^*−*^ and *Eo* ^*+*^ cells, highlighted in red. Importantly, when we directly compared heart enrichment in *Eo* ^*−*^ and *Eo* ^*+*^ cells populations, we found that non-annotated lncRNAs exhibited greater heart enrichment than that of PCGs and Ensembl lncRNAs (Fig. 2b, c). These findings suggest that non-annotated lncRNAs enriched in *Eo* ^*+*^ cells may mediate important regulatory functions for cardiogenic differentiation. To further explore tissue specificity, we repeated this enrichment analysis individually for the eleven non-cardiac tissues derived from mesoderm, endoderm, and ectoderm, and directly compared each individual score to that found for the heart (Fig. 2d). Strikingly, the heart is preferentially associated with *Eo* ^*+*^-enriched previously non-annotated lncRNAs, discovered within the frame of this study, whereas this is not the case for PCGs and Ensembl lncRNAs. These findings emphasize again the exquisite cell and tissue-specificity of non-annotated lncRNA expression, and support the notion that these noncoding transcripts represent important cardiogenic factors. An example of an *Eo* ^*+*^ cell-enriched transcript that is exclusively expressed in the adult heart as compared to other adult tissues is given in Fig. 2e. Mesendodermal transcripts presenting such a pattern of expression might therefore be involved in the maintenance of cardiac identity in the adult heart.

### Enhancer landscape remodeling during mesendoderm specification

**Fig. 3:**
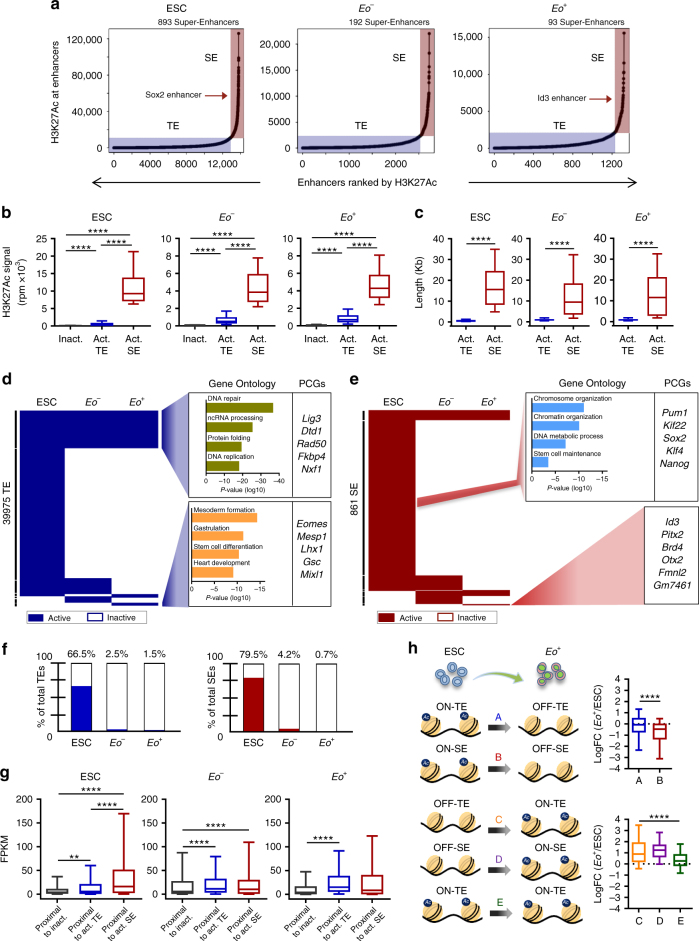
Characterization of the enhancer landscape during mesendoderm specification. **a** Distribution of H3K27Ac identifies TEs and SEs in ESC, *Eo* ^*−*^ and *Eo* ^*+*^. *Sox2* SE and *Id3* SE are indicated as representative SEs **b** H3K27Ac signal (normalized by length) at constituent enhancers within TEs and SEs. Box plot whiskers show median value and 10–90 percentiles. *p* values were calculated using a one-way ANOVA test. (****P < 0.0001). **c** Length (Kb) of active TE and SE regions in ESC, *Eo* ^*−*^ and *Eo* ^*+*^. Median value and 10–90 percentiles are represented. *p* values were calculated using a two-tailed t test. (****P < 0.0001). **d** Heatmap showing active (blue) and inactive (white) TEs across ESC, *Eo* ^*−*^ and *Eo* ^*+*^. GO terms linked to biological processes of PCGs associated to TEs shared in all three conditions and TEs uniquely active in *Eo* ^*+*^ are shown. Examples PCGs are shown to the right. **e** Heatmap showing active (dark red) and inactive (white) SEs across ESC, *Eo* ^*−*^ and *Eo* ^*+*^. GO terms linked to biological processes of PCGs associated to SEs uniquely active in ESC are shown. Examples PCGs in this cluster are shown to the right. The PCGs associated to the six SEs uniquely active *Eo* ^*+*^ are shown. **f** Percentages of uniquely active TEs and SEs in ESC, *Eo* ^*−*^ and *Eo* ^*+*^. **g** Box plot whiskers of PCG expression (FPKM) of genes proximal to inactive TEs, active TEs and active SEs in ESC, *Eo* ^*−*^ and *Eo* ^*+*^. Box plot whiskers show median value and 10–90 percentiles. *p* values were calculated using a one-way ANOVA test. (****P < 0.0001). **h** Schematic of the transition from ESCs to *Eo* ^*+*^. Cluster A: PCGs associated to TEs switched-off during the transition. Cluster B: PCGs associated to SEs switched-off during the transition. Cluster C: PCGs associated to TEs switched-on during the transition. Cluster D: PCGs associated to SEs switched-on during the transition. Cluster E: PCGs associated to TEs active during the transition. Box plot whiskers with LogFC of expression between *Eo* ^*+*^ and ESCs are shown to the right. Median value and 10–90 percentiles are represented. *p* values were calculated using a two-tailed t test. (****P < 0.0001)

Enhancer elements are marked by H3K27Ac, H3K4me1 and by Mediator occupancy. Since these marks largely overlap in previous ChIP-Seq studies^10^, we used H3K27Ac for the identification of enhancers in pluripotent ESC and in *Eo* ^*+*^ and *Eo* ^*−*^ cells. To identify SEs, we used the ROSE algorithm^10^, which stitches together individual enhancers within 12.5Kb of each other to identify a single continuous genomic locus. Stitched enhancers with a H3K27Ac value above a cut-off defined as the point where the slope of the distribution plot of H3K27Ac ChIP-Seq intensity is 1 are designated SEs^10^ (Fig. 3a). Using this distribution plot all remaining genomic loci with ChIP-Seq signal intensity below 1 are classified as TEs. Examples of TE and SE regions are depicted in Supplementary Fig. 5a, b, respectively. As expected, SEs were globally more enriched with H3K27Ac as compared to TEs (Fig. 3b) and spanned much larger genomic regions (Fig. 3c).

To dissect the enhancer state transition that governs mesendodermal gene expression programs, we clustered TE and SE according to their activity across the three cell populations (Fig. 3d, e; Supplementary Fig. 5c, d). A larger set of both TEs and SEs was active in pluripotent ESCs than in the two committed populations. Consistent with previous observations^32^, a gradual restriction in enhancer usage during differentiation was observed, with *Eomes*-fated cells exhibiting the smallest enhancer repertoire (Supplementary Fig. 5e, f). Moreover, a small percentage of TEs and SEs were uniquely active in either *Eo* ^*+*^ or *Eo* ^*−*^ cells (Fig. 3f). Next, we performed a GO analysis of PCGs adjacent to TEs and SEs in each of the three subpopulations (Supplementary Data 4). TEs specific to *Eo* ^*+*^ cells were associated with PCGs linked to relevant terms such as mesoderm formation and heart development (Fig. 3d; Supplementary Fig. 5g). Interestingly, active enhancers in this cell population were adjacent to master regulators of ME and cardiac mesoderm specification, such as *Eomes* and *Mesp1*. SEs identified in ESCs were linked to key regulatory TFs associated with pluripotency, in particular *Sox2* and *Klf4*. Moreover, six SEs were found uniquely activate in *Eo* ^*+*^ cells. Although this small number did not allow a GO analysis to be performed, PCGs adjacent to these SE were master lineage determining TFs such as *Id3*, *Pitx2*, and *Otx2* (Fig. 3e; Supplementary Fig. 5h). We then quantified the expression of PCGs proximal to either TEs or SEs across the three cell populations. In ESCs, PCGs proximal to SEs were typically more expressed than TE-associated PCGs (Fig. 3g). In addition, we investigated the dynamic changes in PCG expression during cell fate determination by measuring expression of PCGs adjacent to TEs or SEs modulated during ESC specification in to *Eo* ^*+*^ cells (Fig. 3h). Interestingly, the transition from ESC to mesendodermal specification was accompanied by a greater downregulation in expression of PCGs linked to inactivated SEs than to inactivated TEs (Fig. 3h; top panel). Finally, a significant activation in expression was observed for PCGs associated to TEs and SEs inactive in ESCs and activated in *Eo* ^*+*^ cells (Fig. 3h; bottom panel). Altogether, these results demonstrate that dynamic remodeling at enhancer loci during ME specification is accompanied by a corresponding modulation of cognate PCGs.

**Fig. 4:**
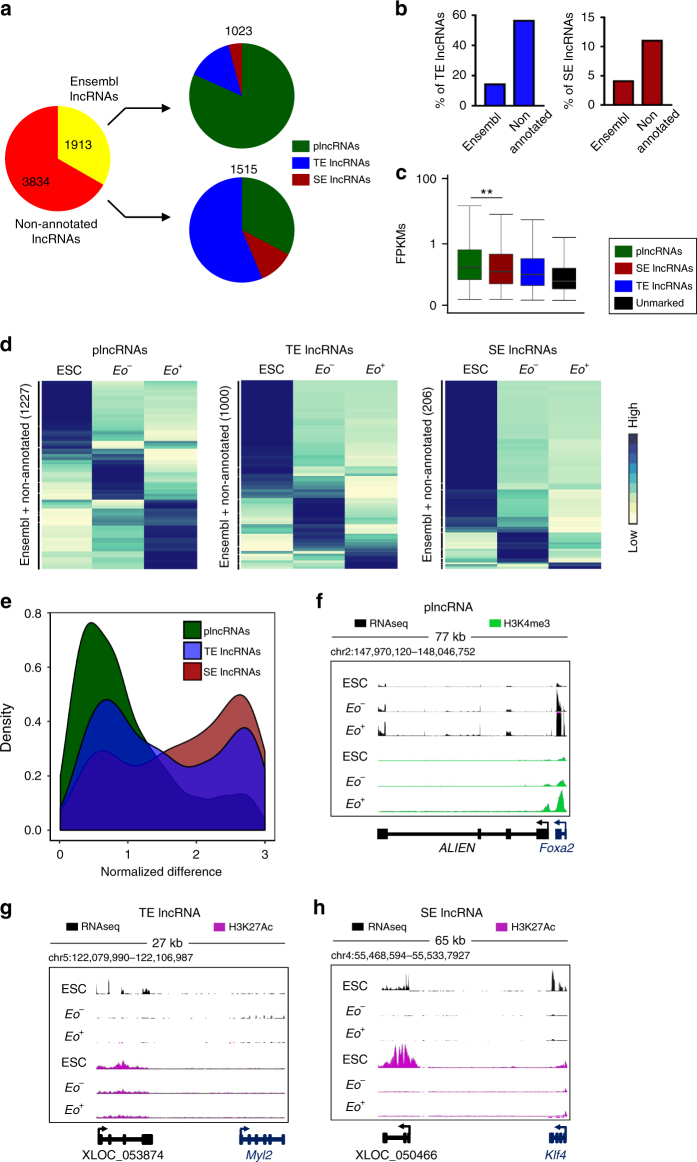
Identification and characterization of promoter, typical and super enhancer associated long noncoding RNAs during mesendoderm specification. **a** Pie chart showing distribution of Ensembl (yellow) and non-annotated (red) lncRNAs associated with a canonical promoter signature (H3K4me3, green), typical enhancer signature (blue) or super enhancer signature (dark red). **b** Percentage of TE lncRNAs (blue) and SE lncRNAs (dark red) in Ensembl and non-annotated lncRNAs. **c** Box plot whiskers of transcript abundance (FPKM) of plncRNAs (green), SE lncRNAs (dark red), TE lncRNAs (blue) and unmarked lncRNAs (black). Box plot whiskers show median value and 10–90 percentiles. *p* values were calculated using a two-tailed t test. (**P < 0.01). **d** Hierarchical clustering of plncRNA, TE lncRNA and SE lncRNA expression across ESC, *Eo* ^*−*^ and *Eo* ^*+*^. **e** Kernel density plot of the specificity of plncRNAs, TE lncRNAs and SE lncRNAs assessed by quantifying the Normalized difference of expression in the three conditions (ESC, *Eo* ^*−*^ *, Eo* ^*+*^). **f** RNAseq and H3K4me3 reads in ESCs, *Eo* ^*−*^ and *Eo* ^+^ cells for example plncRNA (*ALIEN*). **g** RNAseq and H3K27Ac reads in ESCs, *Eo* ^*−*^ and *Eo* ^+^ cells for example TE lncRNA (*XLOC_053874*). **h** RNAseq and H3K27Ac reads in ESCs, *Eo* ^*−*^ and *Eo* ^+^ cells for example SE lncRNA (*XLOC_050466*)

Enhancers are typically associated with the production of lncRNAs, which are believed to contribute to enhancer function. We therefore categorized lncRNAs according to their association with regions marked by either H3K4me3 or H3K27Ac. LncRNAs were classified as being associated with either a canonical promoter signature (H3K4me3, denominated plncRNAs) or an active enhancer (H3K27Ac, TE or SE lncRNAs) (Fig. 4a). Non-annotated lncRNAs were found to be more associated with both TE (60%) and SE (10%) signatures when compared to annotated Ensembl lncRNAs (18 and 4% associated with TE and SE, respectively) (Fig. 4b). Enhancer-associated lncRNAs were expressed at lower levels as compared to promoter-associated lncRNAs (Fig. 4c). We then visualized plncRNA, TE lncRNA and SE lncRNA expression across the three cell populations (Fig. 4d; Supplementary Data 5). LncRNA expression segregates in defined cell-specific patterns. Moreover, the number of cell-specific enhancer-associated lncRNAs expressed in *Eo* ^*+*^ cells, both TE lncRNAs and SE lncRNAs, is much lower than in the two other populations. Interestingly, both TE and SE associated lncRNAs exhibit greater cell-type specificity as compared to plncRNAs (Fig. 4e). Examples of the three classes of lncRNAs are depicted in Fig. 4f–h. These highly specialized expression profiles support specific transcriptional functions for enhancer-associated lncRNA loci during cell-fate determination and differentiation.

### Mesendoderm-specifying loci in pluripotent ESCs

**Fig. 5:**
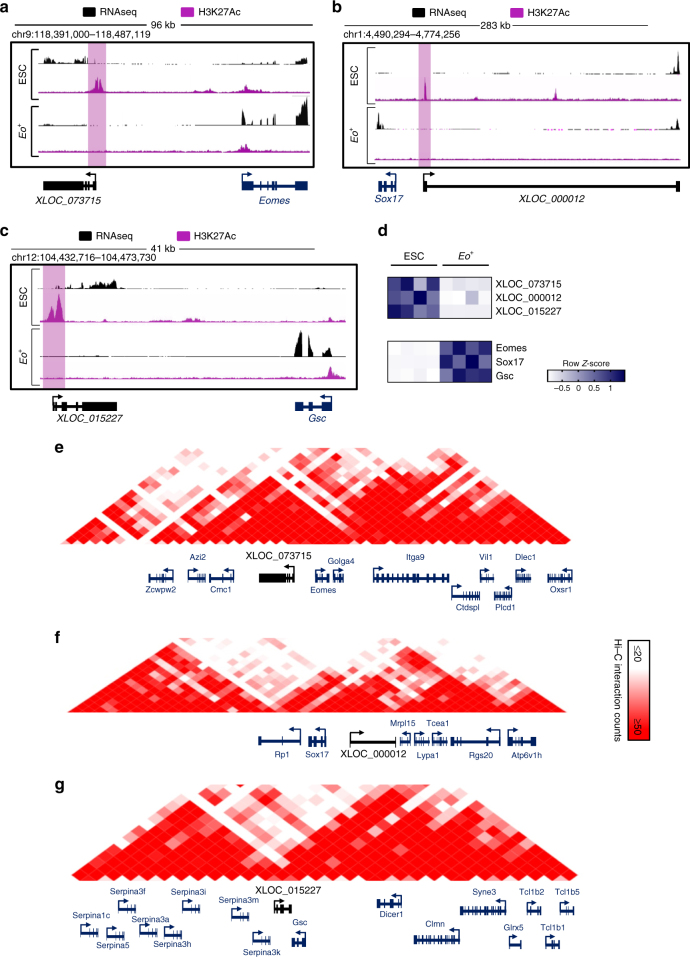
Pluripotency specific enhancer associated lncRNA loci interact with mesendodermal transcription factors. **a** RNAseq and H3K27Ac reads in ESCs and *Eo* ^+^ for *Eomes* and the lncRNA *XLOC_073515*. **b** RNAseq and H3K27Ac reads in ESCs and *Eo* ^+^ for *Sox17* and the lncRNA *XLOC_000012*. **c** RNAseq and H3K27Ac reads in ESCs and *Eo* ^+^ for *Gsc* and the lncRNA *XLOC_015227*. **d** Heatmap showing expression of three lncRNAs candidates (*XLOC_073515*, *XLOC_000012*, and *XLOC_015227*) and their cognate PCGs (*Eomes*, *Sox17,* and *Gsc*) in ESCs and *Eo* ^+^. **e** Hi–C interaction density heatmap of a genomic region where *Eomes* and lncRNA *XLOC_073715* are centered. **f** Hi–C interaction density heatmap of a genomic region where *Sox17* and lncRNA *XLOC_000012* are centered. **g** Hi–C interaction density heatmap of a genomic region where *Gsc* and lncRNA *XLOC_015227* are centered

In addition to the enhancer landscape, chromatin structure and topology is emerging as a critical regulatory feature in pluripotent stem cells that subsequently dictates cell-fate determination and lineage specification^17^. Recently, it emerged that lncRNAs associated with *Cis*-regulatory sequences could play important roles in the nuclear organizing processes that dictate cell fate^18^. We hypothesized that enhancers associated with lncRNAs expressed specifically in pluripotent ESCs, and distal to mesendodermal TFs, may represent critical functional elements dictating developmental competence and ultimately ME specification. We manually selected three mesendodermal TFs, namely *Eomes*, *Sox17* and *Gsc*, based on their location adjacent to an active enhancer expressing an associated lncRNA exclusively in pluripotent ESCs (Table 1). Interestingly, both the enhancer activity, marked by H3K27Ac, and the expression of the associated lncRNA were specific to the ESC stage, and neither the enhancer nor expression of the lncRNA was activated in *Eo* ^*+*^ cells (Fig. 5a–d). Conversely, the adjacent TFs were lowly expressed in ESCs and significantly upregulated in *Eo* ^*+*^ cells. At the pluripotent stage, cell-type invariant topologically associating domains (TAD) are established, which are critical for configuring the chromatin structure ensuring correct temporal and spatial communication between distal enhancers and their cognate cell-fate determining TFs. We thus utilized publicly available high-throughput conformation capture (Hi-C) datasets from murine ESCs to interrogate the topological nature of the different loci containing *Eomes*, *Sox17,* and *Gsc*. All three regions were encompassed within highly interacting chromatin domains (Fig. 5e–g). These data indicated the presence in pluripotent ESCs of chromatin loops that place the enhancers and the mesendodermal TF promoters in close proximity within the three-dimensional nuclear architecture. Altogether, this suggested these loci could play fundamental roles in subsequent *Cis-*regulatory events occurring during ME lineage specification.

**Table 1:** Pluripotency enhancer-associated lncRNA loci distal to ME TFs

Mesendoderm PCGs	Enhancer expressing an associated lncRNA exclusively in ESCs
*Tbx3*	NO
*Gsc*	YES
*Eomes*	YES
*T*	NO
*Mixl1*	NO
*Foxa2*	NO
*Sox17*	YES
*Bmp4*	NO
*Kdr*	NO
*Cxcr4*	NO
*Pdgfr-a*	NO
*Evx1*	NO
*Lhx1*	NO

### The *Meteor* locus dictates mesendoderm specification

**Fig. 6:**
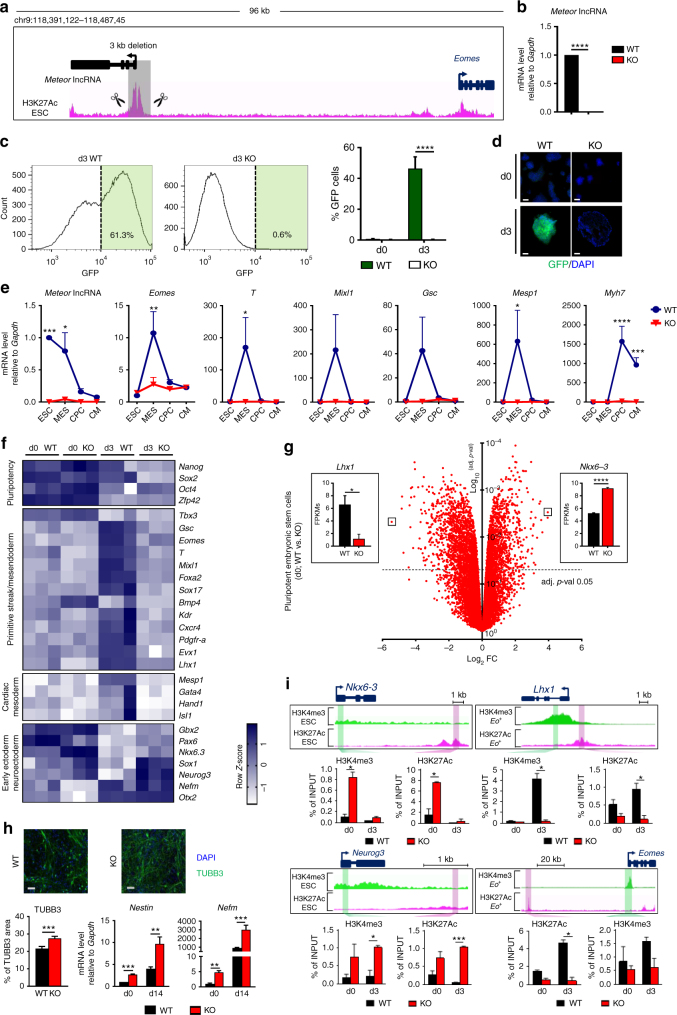
The *Meteor* locus controls the cell fate decision between mesendoderm and neuroectoderm. **a** Genomic deletion in *Meteor* KO cells. **b** Expression of *Meteor* lncRNA in WT and *Meteor* KO ESCs. Bars represent average of fold change normalized to WT. *p* value was calculated using a two-tailed t test. (****P < 0.0001). **c** GFP profile at day 0 and day 3 of differentiation in WT and *Meteor* KO. Percentage of GFP^+^ cells at day 0 and day 3 in WT and *Meteor* KO are shown to the right. Bars represent average of GFP + cells. *p* values were calculated using a two-tailed t test. (****P < 0.0001). **d** Anti-GFP antibody staining on day 0 colonies and day 3 EBs in WT and *Meteor* KO (GFP: green; DAPI: blue). Scale bar represents 50 μm. **e** Expression of *Meteor* lncRNA, *Eomes*, *T*, *Mixl1*, *Gsc*, *Mesp1* and *Myh7* in WT and KO cells during cardiogenic differentiation. Trends represent average of fold change normalized to WT ESC. *p* values were calculated using a two-way ANOVA test. (*P < 0.05; **P < 0.01; ***P < 0.001; ****P < 0.0001). **f** Heatmap of expression of representative PCGs in d0-d3 WT and *Meteor* KO cells. **g** Volcano plot representation of expressed PCGs in d0 WT and d0 *Meteor* KO. FPKM values of *Lhx1* and *Nkx6-3* are shown. Bars represent mean expression ± SEM. *p* values were calculated using a two-tailed t test. (*P < 0.05; ****P < 0.0001). **h** TUBB3 staining in neuronal-like day 14 WT and *Meteor* KO cells. (TUBB3: green; DAPI: blue). Scale bar represents 50 μm. Percentage of the TUBB3 positive area is shown below. Bars represent average of percentage. *p* value was calculated using a two-tailed t test. Expression of *Nestin* and *Nefm* in d0-d14 WT and *Meteor* KO cells. Bars represent average of fold change normalized to day0 WT. *p* values were calculated using a two-tailed t test. (**P < 0.01; ***P < 0.001). **i** H3K27Ac and H3K4me3 enrichment at enhancers and promoters of *Nkx6.3, Lhx1, Neurog3 and Eomes* in d0-d3 WT and *Meteor* KO cells. Bars represent average of percentage of input. *p* values were calculated using a two-tailed t test. (*P < 0.05; ***P < 0.001)

To evaluate the importance of the identified loci for mesendodermal cell fate determination, we selected the enhancer locus upstream of *Eomes* for a detailed analysis. We named the enhancer *Meteor* for MesEndoderm Transcriptional Enhancer Organizing Region. The enhancer was highly active in ESCs, coupled with high expression of its associated lncRNA, while *Eomes* was not significantly expressed at this pluripotent stage. Conversely, *Eomes* was enriched and the *Meteor* lncRNA was depleted in *Eo* ^*+*^ cells (Supplementary Fig. 6a, b). The expression kinetics during cardiogenic differentiation confirmed the downregulation of the *Meteor* lncRNA during differentiation concomitant with the transient induction of *Eomes* (Supplementary Fig. 6c). *Meteor* was bound in pluripotent ESCs by Oct4 and Sox2 but not Nanog (Supplementary Fig. 6d). To probe the molecular and cellular function of this locus, we utilized CRISPR-Cas9 to delete the *Meteor* enhancer in *Eomes* ^EGFP^ ESCs (Fig. 6a). The deleted fragment included the transcriptional start site of the *Meteor* lncRNA (Supplementary Fig. 6e), and resulted in a complete loss of *Meteor* lncRNA expression (Fig. 6b). Importantly, *Meteor* knockout (KO) cells exhibited normal expression of pluripotency markers such as NANOG*/*SSEA-1, and high alkaline phosphatase activity, demonstrating that these cells maintain stemness properties comparable to wild-type (WT) ESCs (Supplementary Fig. 6f–h). *Meteor* KO cells were next analyzed following induction of differentiation. On day 3, approximately fifty percent of wild-type ESC-derived cells expressed EGFP as assessed by flow cytometry (Fig. 6c). In sharp contrast, no EGFP expression was detected in differentiating *Meteor* KO cells (Fig. 6c, d). Furthermore, cell surface analysis of PDGFRα expression, a marker of early mesodermal cells, demonstrated the lack of mesodermal specification during differentiation of *Meteor* KO ESCs (Supplementary Fig. 6i). Consistent with the observed phenotype, differentiating *Meteor* KO ESCs exhibited a major transcriptional defect in the induction of mesendodermal PCGs (*Eomes*, *T*, *Mixl1*, *Gsc*, *Foxa2*, *Lhx1*), and consequently of cardiac mesoderm (*Mesp1*), cardiac precursor (*Gata4*, *Nkx2-5, Mef2c*) and cardiomyocyte (*Myh7*) PCGs (Fig. 6e; Supplementary Fig. 6j).

To gain deeper molecular insights into the mechanisms governing this lineage-specifying defect, we performed RNA-Seq on WT and *Meteor* KO ESCs at day 0 (undifferentiated state) and day 3 of differentiation (Supplementary Data 6). Compared to controls, 1267 PCGs were differentially expressed in *Meteor* KO cells of which 871 were downregulated and 396 upregulated (Supplementary Fig. 7a). Furthermore, at day 3, 1667 PCGs were modulated of which 1101 were downregulated and 566 upregulated (Supplementary Fig. 7b). Core pluripotency factors were not impacted in *Meteor* KO ESCs (Fig. 6f). However, key regulators of primitive streak and ME were strikingly downregulated in differentiating *Meteor* KO cells. As a result, markers linked to cardiac mesoderm were also downregulated (Fig. 6f; Supplementary Fig. 7c). Consistent with this observation, GO analysis of PCGs downregulated in *Meteor*-deleted cells at day 3 of differentiation revealed terms linked to gastrulation and mesoderm formation, supporting that these biological processes are primarily affected in *Meteor*-deficient cells (Supplementary Fig. 7b; Supplementary Data 7). Importantly, *Lhx1* and *Nkx6-3* were respectively the most downregulated and upregulated PCGs in *Meteor*-KO ESCs at day 0 (Fig. 6g). These data indicate *Meteor* deletion during pluripotency is associated with a global transcriptional reprogramming favoring an ectodermal fate while blocking subsequent mesendodermal specification. Accordingly, *Mesp1* was the most downregulated PCG at day 3 while *Neurog3* was the most upregulated (Supplementary Fig. 7d). As a consequence, *Meteor* KO cells were unable to initiate their cardiogenic program and did not generate any beating EBs (Supplementary Fig. 7e). Considering the topological nature of the *Meteor* locus, PCGs encompassed both within and proximal to the TAD harboring *Meteor* may be impacted by *Meteor* deletion in a *Cis*-dependent manner. We examined the expression of PCGs within a 4 Mb region centered on the *Meteor locus* in wild-type and *Meteor*-KO undifferentiated ESCs. Only two of the twenty-six PCGs within this region were significantly downregulated in deleted cells, specifically *Scn5a* and *Mobp* (Supplementary Fig. 7f). Globally, *Meteor* deletion was not associated with a large scale transcriptional dysregulation of local gene expression. In particular, *Eomes* was not modulated in *Meteor*-KO ESCs.

### Increased neurogenic differentiation in *Meteor*-deleted ESCs

Interestingly, regulatory factors linked to early ectoderm and neuroectoderm were upregulated both at day 0 and day 3 in *Meteor*-KO cells. In particular, *Nkx6-3* was the most upregulated gene in undifferentiated KO cells, and *Neurog3* was the most upregulated PCG at day 3 of differentiation (Fig. 6g; Supplementary Fig. 7d). Early ectoderm markers, i.e. *Otx2*, *Pax6*, *Gbx2*, were also significantly induced upon differentiation in *Meteor*-deleted cells, suggesting that, while losing their ability to be specified towards ME, these cells were redirected towards the ectoderm lineage (Supplementary Fig. 7g). To investigate whether *Meteor*-KO cells could harbor greater propensity for differentiating into ectoderm-derived tissues, WT and *Meteor*-KO cells were differentiated using a neurogenic differentiation protocol. Consistent with the induced ectodermal gene program, *Meteor*-KO cells were able to produce increased numbers of TUBB3 positive neurons (Fig. 6h). Enhanced expression of *Nestin* and *Nefm*, two other markers of neuronal differentiation, demonstrated the increased production of mature neurons. The transcriptional reprogramming leading to a blockade of ME specification and activation of a neuroectodermal gene network in *Meteor*-deleted ESCs suggests that *Meteor* may enact a more global *Trans*-regulatory role, either directly via the lncRNA produced from this locus or indirectly by regulating the expression of nearby genes. We therefore investigated whether reprogramming was associated with modifications on chromatin at specific PCG promoters and distal enhancers (Fig. 6i). ChIP-qPCR was performed against H3K4me3 and H3K27Ac using primers targeting promoters and enhancers of PCGs exhibiting the greatest fold changes between WT and *Meteor* KO cells. For example, for both *Nkx6-3* and *Neurog3*, the promoters and the closest distal enhancers were significantly enriched in H3K4me3 and H3K27Ac respectively in *Meteor*-deficient cells. Conversely, both the promoters and enhancers of the ME TFs, *Eomes* and *Lhx1*, were significantly depleted of these respective marks in *Meteor* KO cells. We also assessed the chromatin state at promoters and enhancers of other relevant ectoderm and ME PCGs and confirmed that significant epigenetic remodeling occurred in the absence of the *Meteor* locus (Supplementary Fig. 7h). Finally, we repeated and validated these findings using an alternative, independently generated, *Meteor*-deleted clone (Supplementary Fig. 8a–e). These results support therefore an important role for *Meteor* during pluripotency in controlling the developmental competence to commit into ME and neuroectoderm.

### RNA-independent *Meteor* function in pluripotency

**Fig. 7:**
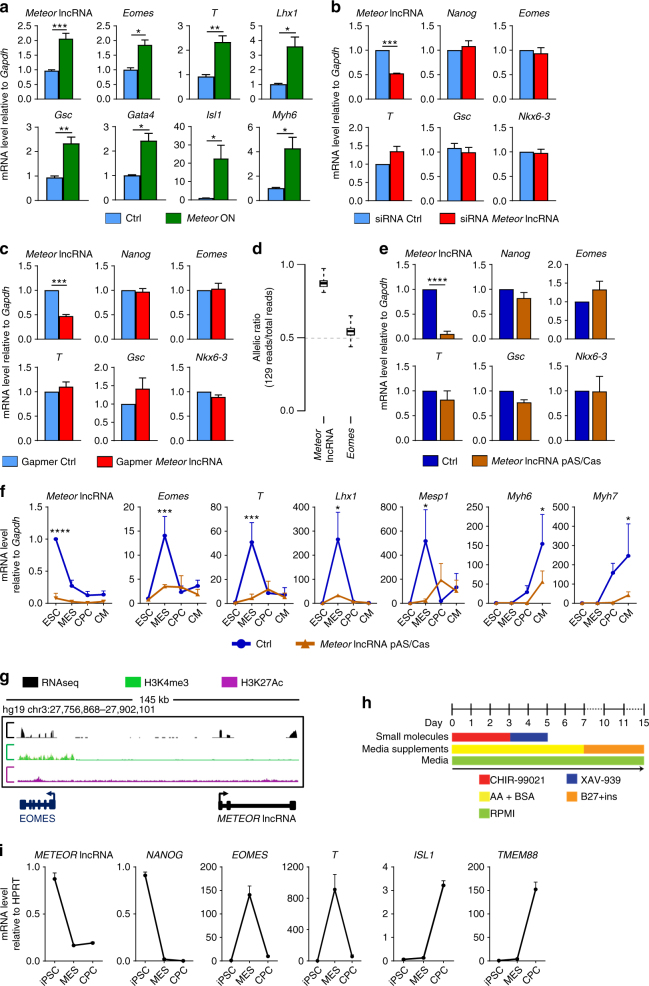
Assessment of *Meteor* functionality. **a** Expression measured by qRT-PCR of *Meteor lncRNA* and ME/Cardiac Mesoderm-related genes in P19CL6 cells following CRISPR-On-mediated *Meteor lncRNA* induction. Mean ± SEM (*n* = 6). *p* values were calculated using a two-tailed t test. (*P < 0.05; **P < 0.01; ***P < 0.001). **b** Expression measured by qRT-PCR of *Meteor lncRNA*, *Nanog*, *Eomes*, *T*, *Gsc* and *Nkx6-3* in ESCs following transfection with siRNAs targeting *Meteor lncRNA* or a control sequence. Mean ± SEM (*n* = 3). *p* values were calculated using a two-tailed *t* test. (***P < 0.001). **c** Expression measured by qRT-PCR of *Meteor lncRNA*, *Nanog*, *Eomes*, *T*, *Gsc* and *Nkx6-3* in ESCs following transfection with GapmeRs targeting *Meteor lncRNA* or a control sequence. Mean ± SEM (*n* = 3). *p* values were calculated using a two-tailed t test. (***P < 0.001). **d** Allelic expression ratios of *Meteor* lncRNA and *Eomes* from targeted RNA sequencing of 63 wild-type 129/Castaneus mESC clones^21^. Box shows interquartile range. Whiskers show min and max. **e** Expression measured by qRT-PCR of *Meteor lncRNA*, *Nanog*, *Eomes*, *T*, *Gsc* and *Nkx6-3* at day 0 in Ctrl (clone transfected with non-targeted guide RNAs) and *Meteor lncRNA* pAS/Cas cells. Mean ± SEM (*n* = 3). *p* values were calculated using a two-tailed t test. (****P < 0.0001). **f** Expression kinetic of *Meteor* lncRNA, *Eomes*, *T*, *Lhx1*, *Mesp1*, *Myh6* and *Myh7* in WT (clone transfected with non-targeting guide RNA) and *Meteor lncRNA* pAS/Cas cells during cardiogenic differentiation measured by qRT-PCR. Trends represent average of fold change (*n* = 3 biological replicates) normalized to Ctrl ESC. *p* values were calculated using a two-way ANOVA test. (*P < 0.05; ***P < 0.001; ****P < 0.0001). **g** RNAseq, H3K4me3 and H3K27Ac reads in human ESCs in the *EOMES* and *METEOR* loci. **h** Schematic illustration of human iPSC cardiac-directed differentiation using small molecular modulation of Wnt signaling. **i** Stage-specific expression kinetic of *METEOR* lncRNA, *NANOG*, *EOMES*, *T*, *ISL1* and *TMEM88* assayed by qRT-PCR. Trends represent average of fold change (*n* = 3 biological replicates) normalized to iPSC

Genetic deletion of the *Meteor* enhancer and its associated lncRNA led to a global transcriptional reprogramming that abrogated the developmental competence of pluripotent ESCs for subsequent ME specification. The functionality of the enhancer can therefore depend on mechanisms that involve the DNA element (e.g., recruiting TFs), the RNA transcript itself and/or processes associated with its production, including the process of transcription and splicing of the transcript. As a first step, we utilized a CRISPR-based gain-of-function approach (CRISPR-On) to boost transcription at the locus and increase *Meteor* lncRNA expression. Embryonic stem cell-like P19CL6 cells were transfected with components of the synergistic activator mediator (SAM)^33^, in combination with a guide-RNA targeting sequences upstream the transcription start site (TSS) of the *Meteor* lncRNA. Induced *Meteor* lncRNA expression was associated with increased expression of key putative downstream ME target genes including *Eomes*, *T*, *Gsc*, in addition to cardiogenic regulators including *Gata4* and *Isl1* (Fig. 7a). This result formally demonstrates that the activation of a pluripotent-specific enhancer such as *Meteor* is sufficient to stimulate downstream pathways controlling cardiogenic differentiation. Of note, early ectoderm and neuroectoderm markers were not affected following *Meteor* activation (Supplementary Fig. 8f). To determine whether *Meteor* enhancer activation was mediated by the *Meteor* lncRNA or was purely a consequence of increased transcription at this locus, we utilized both siRNA and modified antisense oligonucleotides (ASOs) to deplete *Meteor* lncRNA in ESCs (Fig. 7b, c). Both approaches were able to decrease *Meteor* lncRNA levels by ~50%. However, transcript depletion was not associated with perturbed expression of target ME or neuroectodermal genes as observed in *Meteor* KO ESCs (Fig. 7b, c; Supplementary Fig. 8g, h). These data support an RNA-independent mechanism in transcriptional programming of developmental competence occurring in pluripotency.

To confirm these observations, we utilized genetically modified ESC lines engineered to incorporate an early polyadenylation signal (pAS) 1.1 kb downstream of the *Meteor* lncRNA TSS (previously described as linc1405)^21^. Genetic modifications were performed in 129/Castaneus (Cas) F1 hybrid mouse ESCs that contain a polymorphic site every 140 bp, enabling to distinguish allele-specific expression. Importantly, ~90% of *Meteor* lncRNA was transcribed from the 129 allele (Fig. 7d). Insertion of a pAS in the 129 allele (pAS/Cas), therefore, completely abrogated the expression of the *Meteor* lncRNA in engineered ESCs while keeping the *Meteor* enhancer intact (Fig. 7e). Depletion of *Meteor* lncRNA in pluripotent ESCs had no impact on expression of target genes. Altogether, these data demonstrate that the effects of the *Meteor* locus on ME specification in pluripotent cells is RNA-independent and thus likely secondary to enhancer activity.

### Probing *Meteor* functionality during differentiation

We next investigated the importance of *Meteor* lncRNA transcription during ESC differentiation into cardiomyocytes. Upon differentiation, the pAS-modified hybrid cells (pAS/Cas ESCs) lacking *Meteor* lncRNA expression demonstrated a defect in the activation of the ME and cardiogenic gene programs (Fig. 7f). Specifically, the absence of transcript production from the *Meteor* locus abolished expression of key mesendodermal genes such as *Eomes*, *T* and *Lhx1*, and of cardiac mesoderm and cardiac genes, for instance *Mesp1*, *Myh6*, and *Myh7*, resulting in reduced number of beating EBs (Supplementary Fig. 8i). Interestingly, the dynamic downregulation of pluripotency genes were not affected (Supplementary Fig. 8j). Therefore, despite the RNA being dispensable for the programming of ME competence during pluripotency, these data highlighted a dependence on lncRNA transcription during the process of cardiogenic differentiation.

Considering the importance of the *Meteor* locus, we aimed at identifying whether the *Meteor* enhancer was associated with the production of a lncRNA in human (h) ESCs. Publically available RNA and ChIP Seq data suggested transcription at the locus was indeed conserved. As a consequence, the associated transcript (*METEOR* lncRNA) was determined to be highly expressed in hESCs (Fig. 7g). Using an efficient differentiation protocol for ME specification and cardiac differentiation (Fig. 7h; Supplementary Fig. 8k)^34^, we next demonstrated that the human *METEOR* lncRNA was dynamically modulated in an evolutionarily conserved manner during cardiogenic differentiation (Fig. 7i). In particular, the *METEOR* lncRNA was downregulated at the mesendodermal stage, coinciding with the upregulation of key ME markers including *EOMES*, *T* and downstream cardiogenic markers such as *ISL1* and *TMEM88*.

## Discussion

The enhancer landscape is pivotal in establishing the developmental competence of pluripotent ESCs, in particular during the response to inductive signals for ME specification^7,13^. Enhancers are also emerging as key determinants governing both cell fate and identity during the development of the cardiovascular system^1,9^. In this context, the small subset of enhancers associated with the production of multiexonic lncRNAs is of particular importance during commitment to specific fates^14,15^. We therefore disregarded bidirectional eRNAs in the present study. Indeed, enhancers producing multiexonic and polyadenylated lncRNAs exhibit greater chromatin accessibility and are associated with increased binding of key developmental TFs^14^. They are also more likely to elicit formation of promoter-enhancer loops and show greater enhancing activity on target PCGs than non-transcribed enhancers^14^. LncRNA loci are unique in their ability to spatially amplify regulatory information encoded by their underlying DNA^19^. Based on these observations, we characterized the activity of enhancers, profiled the lncRNA transcriptome during ME formation, and finally identified the *Meteor* locus encompassing an enhancer element lying upstream the *Eomes* gene. The *Meteor* enhancer appears crucial to control cell fate determination between the ME and the neuroectoderm, and is essential for cardiac differentiation. An important finding is the fact that the mesendodermal competence of ESCs, for which *Meteor* seems indispensable, is hardwired in pluripotency. These findings are summarized visually in Supplementary Fig. 9.

ChIP-Seq analysis allowed us to map the TE and SE landscapes during mesendodermal commitment. Both TEs and SEs undergo significant restriction during cell fate determination and differentiation. Furthermore, a concomitant activation of a small number of lineage-specific enhancers is observed. This is compatible with a gradual narrowing of the spectrum of cell-fate competence^32^. Importantly, ME-specific TEs were linked to PCGs associated with relevant biological processes such as gastrulation and mesoderm formation. Only six SEs were found uniquely activated in mesendodermal cells. Although this number reflects in part the smaller number of SEs as compared to TEs, this finding is consistent with SEs representing a limited but crucial class of *Cis*-regulatory elements that defines cell identity^10^. Along this vein, global SE activation was associated with significantly greater changes in target PCG expression during differentiation, supporting an important role in controlling dynamics of gene expression. Transcriptomic profiling revealed that the enhancer landscape was associated with the transcription of thousands of multiexonic lncRNAs. This large number of non-annotated transcripts is in part a result of the extreme depth of sequencing utilized in this study^30^. Nevertheless, non-annotated lncRNAs demonstrated interesting characteristics when compared to previously annotated transcripts. In particular, they exhibited more restricted and specialized expression patterns. This is likely a consequence of their enriched association with stage-specific TEs and SEs. Importantly, both TE and SE-associated lncRNAs exhibited significant lineage restriction of expression during differentiation, while plncRNAs exhibited less restricted profiles. SE-associated lncRNAs displayed the greatest restriction, suggesting again such transcripts may mediate important roles during specification and differentiation. Several lncRNAs associated with SEs, including *Carmen* ^35,36^, *CCAT-1L* ^37^, *MyoD-eRNA* ^38^, and *Wisper* ^39^, have recently emerged as important modulators of cell fate determination and maintenance of cell identity, notably in the heart. Globally, non-annotated lncRNAs expressed in mesendodermal precursors were shown to be enriched in the adult heart. The model used to induce ME specification, which favors cardiac differentiation and not definitive endoderm, may partially explain this characteristic. Nevertheless, a significant fraction of the identified lncRNAs may mediate specialized functions in cardiac homeostasis. We also observed a significant difference in the evolutionary characteristics of non-annotated lncRNAs expressed in ME-specified cells. Conservation at promoter regions of those lncRNA loci was greater than that of lncRNAs expressed in other cellular intermediates during cardiogenic differentiation, and greater than that measured in cells not committed to ME. ME formation is a key evolutionary branch point that is represented by *Eo* ^*+*^ cells in our experiments. This point has been termed the phylotypic stage and has given rise to the hourglass model of development^27^. Interestingly, our evolutionary analyzes are in accord with this model and indicate that the hourglass phenomenon may be associated with selection of discrete sets of enhancer-associated lncRNA promoters.

Developmental competence is typically mediated by chromatin states at lineage specifying enhancer and promoter loci^40^. In this context, transcribed enhancers are emerging as key elements for modulating chromatin architecture, in particular as regulators of TAD formation^18,19^. TADs are typically established in ESCs and critical for developmental competence and subsequent germ layer specification^17^. We identified three ESC-specific enhancer associated lncRNA loci highly interacting in three-dimensional space with key ME-specifying TFs, and examined the functional requirements of one specific locus, *Meteor*, containing a transcribed enhancer highly interacting with the *Eomes* promoter. Deleting this single genomic locus in ESCs led to the complete abolition of *Eomes* expression, ME specification and subsequent differentiation into the cardiomyocyte fate. *Eomes* has been previously deleted in ESCs and resulted in perturbed ME specification^8^. However, *Eomes*-deleted cells do not exhibit absolute lack of ME formation. Some ME-specifying TFs such as *Gsc* and *T* are not impacted by *Eomes* deficiency while *Meteor* deletion completely disrupts the mesendodermal gene program. This suggests *Meteor* occupies an upstream position within the GRN regulating ME specification. Importantly, *Meteor*-deleted cells appear to maintain core stemness features suggesting this locus is not required for self-renewal. *Meteor* lncRNA is transcribed from its associated enhancer in a convergent orientation to another enhancer that is activated during ME specification. The importance of this second distal enhancer for *Eomes* expression should be examined in future studies. Interestingly, different proximal enhancers dictating the induction of *Eomes* in the anterior visceral endoderm, primitive streak and definitive endoderm have been recently investigated^41^. A poised preformed chromatin architecture at the *Eomes* locus appears permissive for rapid transcriptional induction in response to nodal signaling during gastrulation via SMAD2/3 binding at these enhancers. The role of *Meteor* and its associated lncRNA in modulating the activity of these proximal enhancers remains to be demonstrated. Nevertheless, based on these results, it is tempting to speculate that this preformed chromatin compartment may be established by *Meteor* during pluripotency. Finally, *Meteor*-deleted ESCs appear to lose their competence for ME specification while maintaining their capacity to produce neuroectodermal lineages. This was already evident at the ESC stage suggesting that *Meteor* is able to epigenetically prime pluripotent cells at key lineage-determining loci prior to commitment into the three germ layers. As a consequence, *Meteor* KO cells give rise to increased number of neurons upon induction of neurogenic differentiation. This is in accordance with previously published studies identifying molecular mechanisms controlling a binary cell fate decision between the mesoderm and the neurectoderm^4,7,42,43^.

Mechanistically, the *Meteor* locus encodes both an enhancer and a lncRNA. Transcription at the locus and/or the RNA itself may be involved in remodeling the local chromatin topology, thereby priming the epigenome for developmental signals^13,19^. However care needs to be taken when interpreting the roles of the enhancer and of its associated lncRNA^44^. A number of transcribed enhancers have been shown to mediate multifunctional roles. For instance, the *Haunt* ^45^, *Hand2* ^46^, and *Nanog* ^47^ enhancers have been shown to encode both RNA-dependent and –independent functions. Recently, the role of the *Meteor* lncRNA, also known as *lnc1405*, was dissected in ESCs^21^. In agreement with our results, the lncRNA and its transcription were dispensable for expression of *Eomes* in undifferentiated pluripotent ESCs. Nevertheless, induction of *Meteor* transcription at a pluripotent stage using a CRISPR-On approach is sufficient to promote mesendoderm specification and to stimulate subsequent cardiogenic differentiation. We therefore further evaluated the role of this locus using ESCs engineered to accommodate a pAS element downstream of the *Meteor* lncRNA TSS. This manipulation affects both transcription and production of the *Meteor* lncRNA, while maintaining an active enhancer, and abolishes commitment towards the cardiomyocyte fate. This demonstrates the crucial importance of transcription at the *Meteor* locus during the differentiation process. The *Meteor* enhancer and its lncRNA may act in a similar way to *Evx1-as*, providing a window of opportunity during pluripotency to prime a permissive, yet poised, chromatin state^28^. Specifically, *Meteor* could lock the genome into permissive three-dimensional topologies for interpreting cell fate-determining inductive signals^18^. Therefore, enhancer-associated lncRNA loci exhibiting high frequency topological interactions with key lineage determining TFs appear to be particularly important for the acquisition of developmental competence. In the context of regenerative medicine, enhancer-associated lncRNA loci like *Meteor* represent ideal targets for manipulating the fate of ESCs and facilitate the production of highly enriched cell populations.

## Methods

### Culture and differentiation of mouse ES cells

Mouse embryonic Eomes^EGFP^ reporter stem cell line was a kind gift of Elizabeth Robertson (University of Oxford, UK)^20^. Cells were cultured on mouse embryonic fibroblast feeders in standard ES cell medium, which consisted of DMEM high-glucose (Life Technologies #31966-021), supplemented with 20% Fetal Bovine Serum (FBS) (Invitrogen #16141079), 1% Non-Essential Amino Acids (Life Technologies 11140-035), 0.1 mM β-mercaptoetanol (Life Technologies #31350-010), Gentamicin (1:500, Gibco #15750-037) and 1000U ml^*−*1^ of Leukemia Inhibitor Factor (ESGRO #ESG1107). Cardiac differentiation of ES cells was induced by aggregating aliquots containing ~ 1000 cells in hanging drop of 25 µl of differentiation medium: IMDM (Life Technologies #21980-032) medium supplemented with 200mM L-glutamine (Life Technologies #25030-024), 20% FBS, 6.5 µl of 1-thioglycerol (Sigma #M6145), 1 M L-ascorbic acid (Sigma #A4544) and Gentamicin (1:500) to form embryoid bodies. At day 3 of differentiation, EBs were collected and transferred into 10 cm non-adherent bacterial petri dishes for growing in suspension. At day6, EBs were collected and plated on tissue culture dishes coated with 0.1% gelatine for further differentiation until day10.

### Immunofluorescence analysis

Cells and/or EBs were fixed for 10 min in 4% paraformaldehyde in PBS and permeabilized with 0.1% Triton × 100 in PBS (Sigma). After blocking in blocking buffer (PBS containing 0.01% Triton × 100 and 1% BSA) they were incubated overnight at 4 °C with the following primary antibodies: chicken anti-GFP antibody (1:1000, Abcam #AB13970), rabbit anti-NANOG antibody (1:500, Abcam #AB80892), mouse anti-βIII-TUBULIN (1:500, R&D Systems), mouse anti-α-ACTININ (1:400, Sigma #7811), rabbit anti-GATA4 (1:200, Abcam #AB134057). The following conjugates antibodies specific to the appropriate species were used: goat anti-Chicken IgY Alexa Fluor® 488 and Alexa Fluor® 500 (1:500, Invitrogen A-11039 and A-21437) and donkey anti-mouse Alexa Fluor® 594 (1:200, Invitrogen A-21203). Nuclei were stained with DAPI (Invitrogen). An Axiovision fluorescence microscope (Carl Zeiss) and a Nikon Eclipse Ti microscope were used for these analyzes.

### Alkaline phosphatase staining

Wild type ESCs and Meteor KO at day 0 were stained for the expression of Alkaline Phosphatase with the Alkaline Phosphatase detection kit (Millipore, #SCR004). An Axioplan microscope (Carl Zeiss) was used for this analysis.

### Flow cytometry

Mouse ES cells and EBs were dissociated using FACS medium and filtered through a 40-μm cell strainer. Live cells were gated on the basis of side scatter, forward scatter and propidium iodide exclusion. Undifferentiated ES cells were gated for the GFP channel to exclude any possible background of GFP signal. ESCs and day 3 cells were analyzed for EGFP expression using the Gallios analyser (Beckman Coulter Life Sciences). Cells obtained from the dissociation of the day3 embryoid bodies were sorted for GFP. A total of 12 × 10^6^ of GFP negative (*Eo* ^*−*^ *)* and GFP positive (*Eo* ^*+*^ *)* cells were sorted (BD FACSAria IIu, BD Biosciences) for performing RNA isolation and Chromatin Immuno Precipitation (ChIP) assay. Day0 WT and day0 *Meteor* KO were analyzed for SSEA-1 expression (1:10, BD #560142) using the Gallios analyser. Day0 WT, day0 *Meteor* KO, day3 WT and day3 *Meteor* KO were analyzed for *PDGFR*α expression (1:10, Miltenyi Biotec #130-102-473) using the Gallios analyser. Flow cytometry analysis was performed on day15 human iPSC-derived cardiomyocytes. Cells were stained using cardiac troponin T antibody (0.2 mg ml^*−*1^, Thermo Fisher, MA5-12960) or the corresponding isotype control (0.5 mg ml^*−*1^, Thermo Fisher, #14-4714-82). Cells were analyzed using a BD FACSCANTO II (Beckton Dickinson,) with FACSDiva software (BD Biosciences). Data analysis for all flow cytometry analyzes was performed using FlowJo (Tree Star).

### RNA extraction, RT-PCR and real-time PCR analysis

Total RNA from cultured cells was extracted using miRNeasy kit (Qiagen) according to the manufacturer’s instructions and quantified with Nanodrop (Thermo scientific). The quality control was performed with bioanalyzer Agilent 2100 (Agilent Technologies). Two steps cDNA synthesis was performed with SuperScript II (Invitrogen), and quantification was carried out using QuantStudio^TM^ 6/7 (Thermofisher). Gene expression was normalized to *Gapdh* and quantified using the ΔΔCt method. Primers or probes used in the manuscript are described in Supplementary Table 1.

### RNA sequencing and analysis in ESC, *Eo*^*−*^ and *Eo*^*+*^

Total RNA was isolated using the RNeasy isolation kit (Qiagen). Sequencing libraries were prepared according to Illumina RNA Seq library kit instructions with Poly(A) selection. Libraries were sequenced with the Illumina HiSeq2000 (2 × 100 bp) using 2 lanes/sample with a multiplex level of 1 (~5 × 10^8^ reads per sample) for a total of twelve samples from four different differentiation sets: four ESC, four *Eo* ^*−*^ and four *Eo* ^*+*^. 100 nt paired-end reads were mapped to mm10 reference genome using STAR software version 2.4.0 g, using Ensembl GRCm38.77 reference genes GTF. An *ab initio* transcript reconstruction was performed using Cufflinks, version 2.2.1^48^. As the RNASeq data is stranded, parameter library-type was set to fr-firststrand. The other parameters were default. The resulting GTFs were merged using Cuffmerge, version 2.2.1^49^, using option –g with Ensembl GRCm38.77 GTF as reference, allowing distinguishing known and non-annotated transcripts. Read counts were calculated per gene from the alignment bam files using HTSeq (v0.6.1) with options -m union --stranded reverse. Genes were then filtered for minimal expression (at least one condition with average > 0.1 FPKM).

### Classification of lncRNA

Using the GTF output of Cuffmerge, the transcripts were classified into 3 categories: known mRNAs, known lncRNAs (using Ensembl as reference) and non-annotated lncRNAs. Non-annotated transcripts were filtered for minimal length of 200 bp and at least 2 exons. lncRNA genes were classified into several categories by comparing the lncRNA exon and gene coordinates with coordinates of known protein coding genes.

### LncRNA analysis

Coding potential: The protein-coding potential of transcripts was evaluated using the program GeneID^50^, version v1.4.4, applied to transcript sequences in FASTA format, with 8 parameters adapted for vertebrates as provided by the authors in file GeneID.human.070123.param, and with options -s and -G.Expression heatmaps and gene ontology analysis: Unsupervised clustering of PCGs, Ensembl lncRNAs and non-annotated lncRNAs was generated using the Euclidian distance between the FPKM values of the genes. The PCG, Ensembl lncRNA and non-annotated lncRNA expression heatmaps were generated by clustering the genes by Pearson correlation of the FPKMs, and clustering using the hclust function (method = ”complete”). Heatmap.2 was used to generate the heatmaps. Values were scaled by row. The clusters were manually rearranged.

Differential expression analysis of lncRNAs: Differentially expressed genes were detected using the limma package in R by first transforming the raw count data to log2-cpm (counts per million reads) using the *voom* function. Empirical Bayes moderated t statistics and corresponding p-values were then computed for the 3 comparisons: *Eo* ^*−*^/ESC; *Eo* ^*+*^ / ESC and *Eo* ^*+*^/*Eo* ^*−*^. *P* values were adjusted for multiple comparisons using the Benjamini Hochberg procedure^51^. Genes with an adjusted *p* value of < = 0.05 were considered to be differentially expressed.

Transcript cell specificity: The specificity of PCGs and lncRNAs was assessed by quantifying the Normalized Difference (ND) of expression in the three conditions (ESC, *Eo* ^*−*^ *, Eo* ^*+*^). The Normalized Difference of a PCG or lncRNA *x* was quantified as the maximum difference between its expression *gx* (FPKM normalized) in the three conditions, divided by its average gene expression (as normalizing factor). Formally:

$$ND\left( x \right) = \frac{{\max \left( {g_x} \right) - \min (g_x)}}{{{\rm mean}(g_x)}}$$

The distributions of the Normalized Difference in the three classes (PCGs, Ensembl lncRNAs and non-annotated lncRNAs) were formally compared to each other using the one-tail Wilcoxon rank sum test. Furthermore, the Normalized Difference was used to measure the specificity of the three classes of lncRNAs (plncRNA, TE lncRNA and SE lncRNA). The density plots of the distribution of normalized difference were generated using the Gaussian kernel density estimator implemented in the R package.

### Analysis of gene conservation

PhastCons: The scores calculated on a multiple alignments of 60 vertebrate genomes to the mm10 mouse genome by chromosome were downloaded from the UCSC website^52^. For each gene, scores per base for exons, introns and promoters (defined as 1000 bp upstream from TSS) were summed and divided by the fragment length. This result was used as the score per fragment. In total 50,000 random intergenic regions were generated (size = 3400 bp ± 20%) and the same score was calculated. Log10 of the scores was plotted by category using R package lattice^53^. The scores of the intergenic regions were calculated as a comparison.

PhyloP: The scores calculated on a multiple alignments of 60 vertebrate genomes to the mm10 mouse genome by chromosome were downloaded from the UCSC website^52^. The details of the 60 vertebrate genomes can be visualized at the following link: http://hgdownload.cse.ucsc.edu/goldenpath/mm10/phastCons60way/. For each transcript, the maximum per base phyloP score was taken over 600 bp (500 bp upstream from the TSS, 100 downstream). The maximum value by transcript was used as value for the gene. In Supplementary Fig. 3d enriched transcripts for ESCs, *Eo* ^*−*^ and *Eo* ^*+*^ are defined from the hierarchical clustering of expression in Supplementary Fig. 4b and Fig. 1e, f. Cardiac Precursor Cells (CPC) enriched transcripts are transcripts significantly overexpressed (adj.pVal < 0.05; fold change > 2) in day6 differentiating ESCs vs. day0 undifferentiated ESCs in our previous study^26^. Adult heart enriched transcripts are transcripts significantly expressed (FPKM > 0.5) in the mouse adult heart in our previous study^54^.

### Gene ontology analysis

Gene Ontology (GO) analysis was performed using GREAT (Genomic Regions Enrichment of Annotations Tool) to analyze biological processes ontology terms^55^. The whole mouse genome was used as background.

### Gene expression across tissues

Expression of the genes (PCGs, Ensembl lncRNA and non-annotated lncRNAs) in 12 mouse tissues (Thymus, Liver, Stomach, Colon, Ovary, Spleen, Heart, Kidney, Mammary gland, Frontal lobe, Cortex, Cerebellum) was measured on ENCODE public data (CSHL Long RNA-seq, PI Gingeras, Lab CSHL-m) (The ENCODE Project Consortium, 2011). Counts on plus and minus strands were summed and mean counts were taken for the two replicates per tissue. Between sample normalization was performed using DESeq estimateSizeFactors function^56^. Only genes with minimal expression were kept (at least 0.1 FPKM in one of the conditions).

Heart Enrichment score (per gene) was defined as:

$${\rm HS}\,{\rm score} = \frac{{\mu _{{\mathrm{cardiac}}}}}{{\mu _{{\mathrm{non - cardiac}} + 2}\times\sigma _{{\mathrm{non - cardiac}}}}}$$

Where μ cardiac is the average expression per gene in ENCODE adult heart tissues, μ non-cardiac is the average expression per gene in the 11 other ENCODE samples^31^, and σ is the standard deviation per gene in non-cardiac ENCODE samples. A gene was considered tissue enriched with enrichment score > 1. The clustering was performed using hclust, version 1.3.1, using Spearman correlation and euclidean distance, average linkage clustering. A scaling by row was applied. The specificity side bars (in red) were generated using the Heart Enrichment score defined above. Heatmaps were generated using heatmap.2 from the package gplots in R, version 2.17.0^57^. The analysis was performed in parallel on subsets of genes, using *Eo* ^*−*^, resp. *Eo* ^*+*^ enriched genes, defined by comparing the expression in the 2 conditions. The filter for differentially expressed genes was an adjusted p-value for differential expression < 0.05 and an absolute value of log Fold Change > 1.

Tissue enrichment score (per gene) was defined as:

$${\rm Tissue}\,{\rm Enrichment}\,{\rm score} = \frac{{\mu _{{\mathrm{tissue}}}x}}{{\mu _{{\mathrm{non - tissue}}}x_{ + 2}\times\sigma _{{\mathrm{non - tissue}}}x}}$$

Tissue enrichment ratio for each of the 12 ENCODE tissues was defined as:

$${\mathrm{Tissue}}\,{\mathrm{Enrichment}}\,{\mathrm{ratio}} = \frac{{{\mathrm{Tissue}}\,{\mathrm{Enrichment}}\,{\mathrm{score}}\,{\mathrm{in}}\,{\mathrm{Eo}}^{+} }}{{{\mathrm{Tissue}}\,{\mathrm{Enrichment}}\,{\mathrm{score}}\,{\mathrm{in}}\,{\mathrm{Eo}}^{-} }}$$

### Chromatin immunoprecipitation (ChIP) assay

Cells were cross-linked with 1% formaldehyde for 10 min at RT. Cross-linking reaction was stopped by addition of 0.125 M glycine for 5 min. Chromatin extracted from 1 × 10^7^ cross-linked cells was sonicated to an average of 200–700 bp with the Covaris sonicator (S220 Focused-ultrasonicator). Chromatin Immunoprecipitation (ChIP) was carried out as following: magnetic dynabeads (Dynabeads Protein G, Novex ref #10004D) were pre-coated with the specific ChIP antibody (Ab) for 4 h in a rotating platform at 4 °C. The following Abs were used: H3K27Ac (2 μg of Ab with 60 μL of magnetic beads in 1 mL of total volume, Abcam #4729) and H3K4me3 (4 μg of Ab with 60 μL of magnetic beads in 1 mL of total volume, Abcam #8580). The fragmented chromatin and the coated beads were incubated ON at 4 °C under 10 RPM rotation. After the IP the samples were washed and treated with Proteinase K (500 ng/μL final) and RNase A (20 mg/mL final) and finally purified with the MinElute PCR Purification Kit (QIAGEN, cat #28006). Immunoprecipitated DNA was quantified by Quibit (Life Technologies) and subject to qPCR (primers used in the manuscript are described in Supplementary Table 2) or high-throughput sequencing analysis. For ChIP-seq experiments, libraries for sequencing were prepared with MicroPlex v2 kit (Diagenode) using 10 ng of Chromatin without size selection from total input chromatin and immunoprecipitated DNA. Libraries were subjected to 50-bp single-end read analysis on an Illumina HiSeq 2500.

### ChIP-sequencing analysis

Calling of Constituent Enhancer (CE) regions: 50 bp paired-end short reads were aligned to the mm10 mouse genome using Bowtie (option --non-deterministic was used, the rest was default). Duplicated reads were removed using picard-tools (V. 1.80), MarkDuplicates function. Sequences were extended to 200 bp and allocated in 25-bp bins. Counts per bin were generated using a custom script. Biological replicate whole cell extracts were sequenced for each time point and combined by time point. A Poissonian model was used to determine statistically enriched bins with a p-value threshold set at 1 × 10^*−*9^ ^58^. In addition, we required that genomic bins were at least five fold over input to be considered enriched peaks. To obtain a score by condition, the bins were marked as positive if both replicates of the condition were positive (intersection). The final list of constituent enhancers was defined as the union of contiguous marked bins from the three conditions (so CE existing in at least 1 condition), provided the size was at least 200 bp long.

Calling of Super Enhancer (SE) and Typical Enhancer (TE) regions: The ROSE software (Version 0.1, April 2013, http://bitbucket.org/young_computation/rose) was then run on each replicate of each condition, using the CE defined by condition. This flagged CEs as SE or TE, for each replicate. The intersection of the 2 replicates was taken in each condition, defining a list of SE per condition. The plots shown in Fig. 3a have been generated by the ROSE software. They show 1 replicate for each of the conditions. Then the union of the SE coordinates in the 3 conditions was taken to define a global list of Super Enhancers. The Typical Enhancers were defined as the Constituent Enhancers not overlapping with the union of Super Enhancers in the 3 conditions.

The ChIPSeq scores per SE and TE were then calculated using the same custom script. The heatmaps of active and inactive H3K27Ac regions (Fig. 3d, e, resp. TE and SE) were generated by taking the overlap (of any length), coordinate-wise, of the global list of enhancers (resp. TE and SE) with the list of each individual condition. If a particular enhancer of one of the conditions overlapped with the union of the enhancers, it was marked as active in this condition. The clusters of the heatmaps were then sorted to have the active in 3 conditions on the top, and the active only in *Eo* ^*+*^ on the bottom. The bigWig tracks used in the UCSC genome browser were generated using the USeq software (V. 8.9.3), Sam2USeq function. It generates per base read depth stair-step graph files for genome browser visualization. The values have been scaled per million mapped reads. The useq files have first been converted to wig, then to bigwig, using USeq2Text and UCSC’s software, wigToBigWig.

### Epigenomic annotation of lncRNAs

The Ensembl and non-annotated lncRNAs were classified as: (1) Promoter associated lncRNA if the region ± 1 kb around the TSS was marked positive for H3K4me3 (using the Poissonian model and ratio over input as described above). (2) Super-enhancer associated lncRNA if the region ± 1 kb around the TSS was not marked as H3K4me3 positive and if any part of the transcript was marked as Super Enhancer. (3) Typical-enhancer associated lncRNA: same as above, but with any part of the transcript was marked as Typical Enhancer. (4) Unmarked lncRNA if no chromatin marked was present on the gene.

### Hi-C data

Hi-C plots were generated using the 3D Genome Browser (http://promoter.bx.psu.edu/hi-c/index.html) using publicly available Hi-C data on mouse ESCs^59^.

### Genetic deletion with CRISPR-Cas9 nickase

gRNA design and production of CRISPR-Cas9 nickase constructs: Guide RNAs (gRNAs) and CRISPR Cas9-D10A nickase were encoded in a modified expression plasmid, pX335 (pX335_G2P) kindly offered by Phillip Grote (Goethe, Universität Frankfurt). pX335-G2P plasmid was digested using BbsI restriction enzyme (Thermofisher, #ER1011) and gRNA sequences were designed using the web resource http://crispr.mit.edu (gRNAs used in the manuscript are described in Supplementary Table 3). Pairs of DNA oligonucleotides harboring variable 20 nucleotide sequences (G + 19 bases) were annealed and ligated into the modified pX335_G2P plasmid using the rapid DNA ligation kit (Roche, #11 635 379 001).

ESC transfection and selection: 300,000 ESCs were plated on gelatinized six-well tissue culture plate the night prior to transfection. The next day, cells were transfected with gRNAs using lipofectamin 2000 reagent (Invitrogen, #11668-019). After 4 h of transfection, mESCs were trypzinised and plated at low density in 10-cm plates and clones grown for 6–7 days in ESC medium supplemented with 2 µg ml^*−*1^ of puromycin (Sigma, #P8833-100MG). Individual ESC clones were picked, expanded and analyzed by PCR genotyping. Primers spanning different regions of the deleted region were designed (Supplementary Table 4).

### Sequencing of RNA isolated from WT and *Meteor* KO cells

Total RNA was isolated from WT d0 (*n* = 3), *Meteor* KO d0 (*n* = 3), WT d3 (*n* = 3) and *Meteor* KO d3 (*n* = 3) using the miRNeasy kit (Qiagen). Sequencing libraries were prepared according to Illumina RNA Seq library kit instructions with PolyA selection. Libraries were sequenced with the Illumina HiSeq2000 (1 × 100 bp). Purity-filtered reads were adapter and quality trimmed with Cutadapt (v. 1.3), and filtered for low complexity with seq_crumbs (v. 0.1.8). Reads were aligned against the Mus musculus.GRCm38.82 genome using STAR^60^ (v. 2.4.2a). The number of read counts per gene locus was summarized with htseq-count^61^ (v. 0.6.1) using Mus musculus.GRCm38.82 gene annotation. Quality of the RNA-Seq data alignment was assessed using RSeQC^62^ (v. 2.3.7). Reads were also aligned to the Mus musculus.GRCm38.82 transcriptome using STAR^60^ (v. 2.4.2a), and the estimation of the isoform abundance was computed using RSEM^63^ (v. 1.2.19). Statistical analysis was performed for genes and isoforms independently in R (R version 3.1.2). Genes/Isoforms with low counts were filtered out according to the rule of 1 count per million (cpm) in at least 1 sample. Library sizes were scaled using TMM normalization^64^ (EdgeR v 3.8.5) and log-transformed with limma voom function (R version 3.22.4). Statistical quality controls were performed through pairwise sample correlations, clustering and sample PCA. Replicates cluster together and are well separated between conditions. Differential expression was computed with limma^65^ by fitting data into a linear model, adding the factor for the batch effect and comparing GapmeR vs. control conditions. The *P*-values were adjusted for multiple comparisons using the Benjamini-Hochberg method^51^, controlling for false discovery rate (FDR) or adjusted *P* value.

### Neuronal differentiation of ESCs

Mouse embryonic Eomes^EGFP^ reporter stem cells (WT and *Meteor* KO) were maintained and differentiated into embryonic stem cell derived neurons (ESNs) in a 37 °C tissue culture incubator at 5% CO_2_^66^. Briefly, cells were maintained on coated tissue culture flasks (0.1% gelatine, Sigma) in ES medium consisting of DMEM with Glutamax, 15% fetal bovine serum, embryonic stem cell-qualified (GIBCO^®^) supplemented with 100x MEM non-essential amino acids, 100x penicillin-streptomycin-glutamin, 2-mercaptoethanol (0.1 mM) as well as leukemia inhibitory factor (LIF) to a final concentration of 1000 U ml^*−*1^ (EmdMillipore). Unless otherwise specified, all cell culture supplies for embryonic stem cell maintenance, ESN differentiation and ESN culture were obtained from Invitrogen. To elicit embryoid body (EB) formation and induce differentiation of the mESCs, 1–2 × 10^6^ cells were plated on day 0 in a 10 cm tissue culture dish and the medium was changed to a differentiation medium (DMNK^+^) consisting of a 1:1 mixture of DMEM-F12/Glutamax and Neurobasal medium, supplemented with ×100 penicillin-streptomycin-glutamin, 2-mercaptoethanol (0.1 mM) and 15% KnockOut^TM^ serum replacement. After 1 day in culture all floating EBs were transferred into a 15 ml tube, discarding all adherent aggregates. EBs were centrifuged at low speed (3 min; 35×*g*), resuspended in 10 ml of DMNK^+^ medium and replated in a new 10 cm dish. On day 2 of differentiation, EBs were supplemented with 1 µM retinoic acid to induce neuralization. The next day, the medium was supplemented with 1 µM of smoothened Agonist (SAG,Emd Millipore). At day 7, EBs were dissociated using 2 ml of Accumax (Emd Millipore) for 10 min at room temperature, diluted with 2 ml of DMNK^+^ medium and carefully pipetted up and down 30 times using a 1000 µl pipette. Cells were filtered through a cell 100 µm strainer (BD Falcon) to obtain a single cell suspension. This step was repeated twice to enrich the yield of single cells. The suspension was then centrifuged (3 min; 35×*g*) and resuspended in 2 ml of DMNK^+^ medium supplemented with GDNF (10 ng ml^*−*1^), BDNF (10 ng ml^*−*1^), CNTF (20 ng ml^*−*1^) (all R&D Systems). In total 2 × 10^5^ cells/ml in DMNK^+^ medium supplemented with factors were then plated onto round cover glasses (HUBERLAB, Ø12 mm) which were previously coated for 30 min at 37 °C with Poly-L-lysine (Sigma) and washed twice with PBS. The cells were then cultured at 37 °C, 5% CO_2_ for 7 days, changing medium every 3 days. The RNA was extracted using miRNeasy kit (Qiagen).

### CRISPR-On assay

CRISPR-based gain-of-function was used to activate *Meteor lncRNA* expression in P19CL6 cells (RCB2318, RIKEN Cell Bank, Japan). In total 2 × 10^5^ cells were plated and cultured in DMEM with 10% FCS and transfected (12 h after seeding) with the following components of the synergistic activation mediator (SAM)^33^: a nucleolytically inactive Cas9-VP64 fusion (Addgene plasmid #61425), a MS2-P65-HSF1 activation helper protein (Addgene plasmid #89308) and the *Meteor lncRNA*-targeting guide RNA engineered to contain two MS2 aptamers (sgRNAMS2; Addgene plasmid #61424). Plasmids were transfected at a 1:1 ratio using Lipofectamine 2000 (Invitrogen #11668-019) according to manufacturer’s instructions. Total RNA was isolated 48 h after transfection using miRNeasy kit (Qiagen) and subjected to qRT-PCR.

### Post transcriptional silencing of *Meteor lncRNA*

In total 2 × 10^5^ mouse ESCs were plated and transfected (24 h after seeding) with 20 nM of a siRNA targeting *Meteor lncRNA*: GUU GCU CCG GUC AGA GGU U or a scrambled siRNA (Thermo Fisher) using RNAimax Lipofectamine (Invitrogen, #13778-150). In total 2 × 10^5^ mouse ESCs were plated and transfected (24 h after seeding) with 100 nM of a GapmerR (Exiqon) targeting *Meteor lncRNA*: ATCATGCCTTAAGTGT or a scrambled GapmeR using Lipofectamine 2000 (Invitrogen #11668-019). For both experiments total RNA was isolated 48 h after transfection using miRNeasy kit (Qiagen) and subjected to qRT-PCR.

### *Meteor lncRNA* polyA stop signal in 129/castaneus hybrid ESCs

We took advantage of a mouse ESC line previously generated in another study^21^. In this cell line, a polyadenylation signal (pAS) is placed 1.1 kb downstream of the Meteor lncRNA transcription start site in the 129 allele. This genetic modification was performed in 129/Castaneus (Cas) F1 hybrid mouse ESCs that contain a polymorphic site every 140 bp, enabling to distinguish allele-specific expression.

### Cardiac differentiation of human induced pluripotent stem cells (iPSCs)

WTC11 human iPSCs were plated on Vitronectin XF (Stemcell Technologies #07180) coated plates and maintained in an undifferentiated state in mTeSR 1 media (Stemcell Technologies #85850)^34^. All experiments using human cells were carried out in accordance with human research ethics committee approval at The University of Queensland (Australia). Small molecule cardiac-directed differentiation using a monolayer platform was performed with a modified protocol based on previous reports^34,67,68^. The differentiation set up was initiated by plating undifferentiated hiPSCs as single cells for 24 h. Cells were induced to differentiate (designated day 0) with RPMI 1640 media (Invitrogen #11875093) containing 3 µM CHIR-99021 (Stemcell Technologies #72054), 213 µg ml^*−*1^ L-ascorbic acid 2-phosphate, and 500 µg ml^*−*1^ bovine serum albumin (all Sigma Aldrich). On day 3, media was changed to RPMI 1640 media with 1 µM XAV-939 (Stemcell Technologies #72674), L-ascorbic acid 2-phosphate, and bovine serum albumin (all Sigma Aldrich). On day 5, media was changed to RPMI 1640 media with L-ascorbic acid 2-phosphate and bovine serum albumin. From day 7 onward media was replaced with RPMI 1640 containing B27 supplement with insulin (Invitrogen #17504001). Total RNA was isolated using the RNeasy Miniprep kit (Qiagen). First-strand cDNA was synthesized using the Superscript III reverse transcriptase kit (Invitrogen). Quantitative RT-PCR was performed using SYBR Green PCR Master Mix (Invitrogen) on a ViiA7 Real-Time PCR System with 384-Well Block (Applied Biosystems). The gene expression for each transcript is relative to that of HPRT. Primers used for quantitative RT-PCR are listed in Supplementary Table 5.

### Statistical analysis

GraphPad Software was used for statistical analysis. Data throughout the paper are expressed as mean ± SEM. Statistical significance between two columns was assessed by two-tailed unpaired Student’s t test; for more than two columns, one-way ANOVA (Fisher’s LSD test) analysis was used. Two-way ANOVA (Fisher’s LSD test) was used to evaluate statistical significance between two or more groups. Correlation analysis was performed with Pearson (R or R2 values; 95% confidence interval) or Spearman (R; 95% confidence interval) test. *P* values < 0.05 were considered significant in all events.

### Data availability

The authors declare that all data supporting the findings of this study are available within the article and its Supplementary Information files or from the corresponding author on reasonable request. The RNA-seq and ChIP-seq data reported in this paper have been deposited in NCBI GEO under the accession code: GSE103263 (ESC, *Eo* ^*−*^ and *Eo* ^*+*^) and GSE103583 (WT vs. Meteor KO).

## Electronic supplementary material

Supplementary Information

Description of Additional Supplementary Files

Supplementary Data 1

Supplementary Data 2

Supplementary Data 3

Supplementary Data 4

Supplementary Data 5

Supplementary Data 6

Supplementary Data 7
