# Supplementary material for: A transcribed enhancer dictates mesendoderm specification in pluripotency
Source: Nat Commun. 2017 Nov 27;8:1806. doi: 10.1038/s41467-017-01804-w (PMC5703900; doi:10.1038/s41467-017-01804-w)
Supplement: Supplementary file 1 — Supplementary Information [file 41467_2017_1804_MOESM1_ESM.pdf]

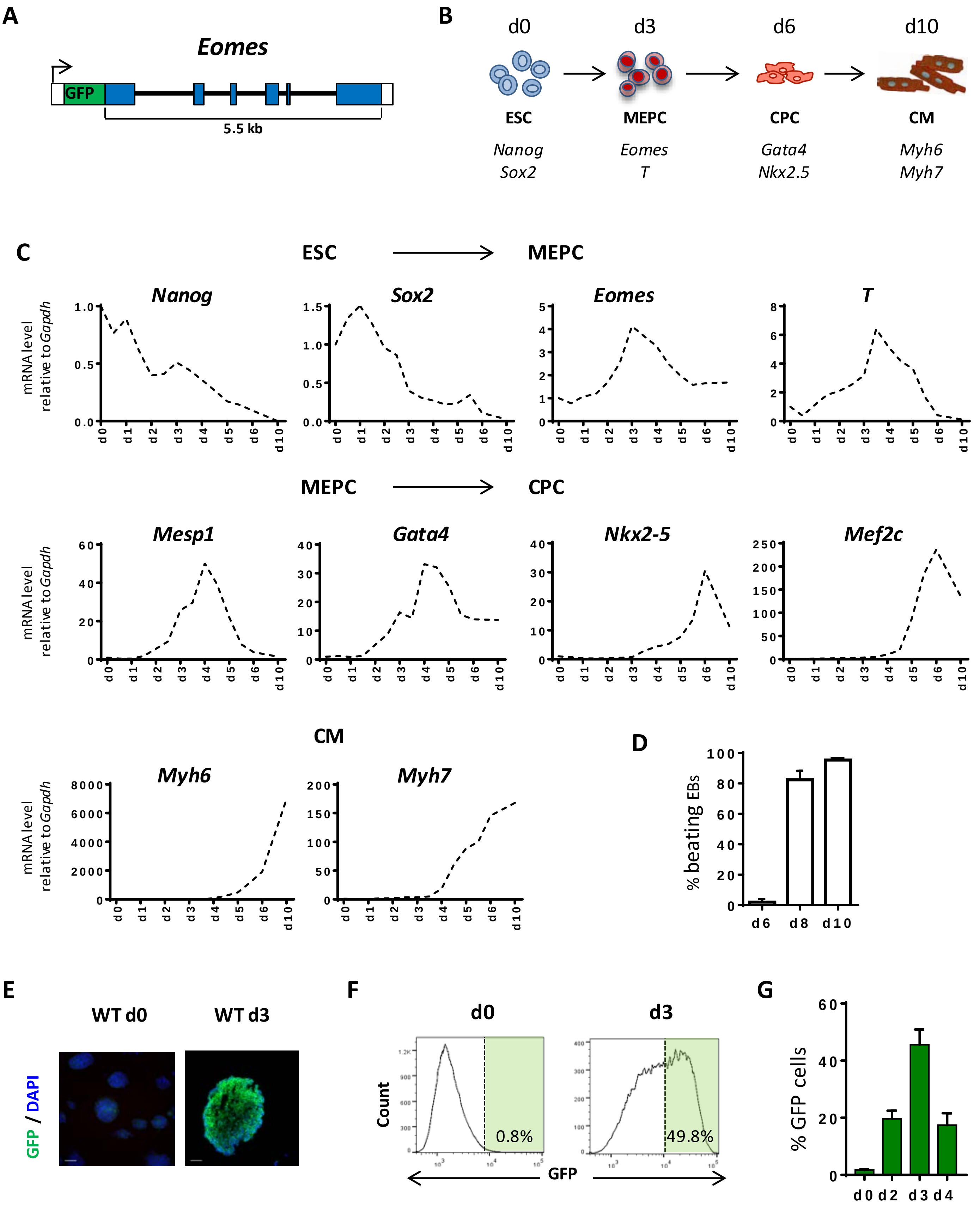


Suppl. Figure 1

**Supplementary Figure 1. Characterization of the *Eomes* reporter mESC line**. (**A**) Schematic of the *Eomes*$^{EGFP}$ ESCs reporter line. (**B**) The four stages of cardiogenic *in vitro* differentiation and marker genes for each stage are given. ESC: Embryonic Stem Cells; MEPC: Mesendoderm Precursor Cells; CPC: Cardiac Precursor Cells; CM: Cardiomyocytes. (**C**) Kinetics of key markers for pluripotency, mesendoderm specification, cardiac mesoderm, CPCs and CMs. Samples were isolated every twelve hours from ESC to day6 and at day10. Trends represent average of fold change (n=3 biological replicates) normalized to day0. (**D**) Percentage of beating embryoid bodies (EBs) at day6, day8 and day10. Bars represent mean ± SEM (n=3). (**E**) Anti-GFP antibody staining on day0 colonies and day3 EBs. (GFP: green; DAPI: blue). Scale represents 50 μm. (**F**) Flow Cytometry (FC) analysis: GFP profile at day0 and day3 of differentiation. (**G**) FC analysis: percentage of GFP$^{+}$ cells at day0, day2, day3 and day4 of differentiation. Bars represent mean ± SEM (n=3).

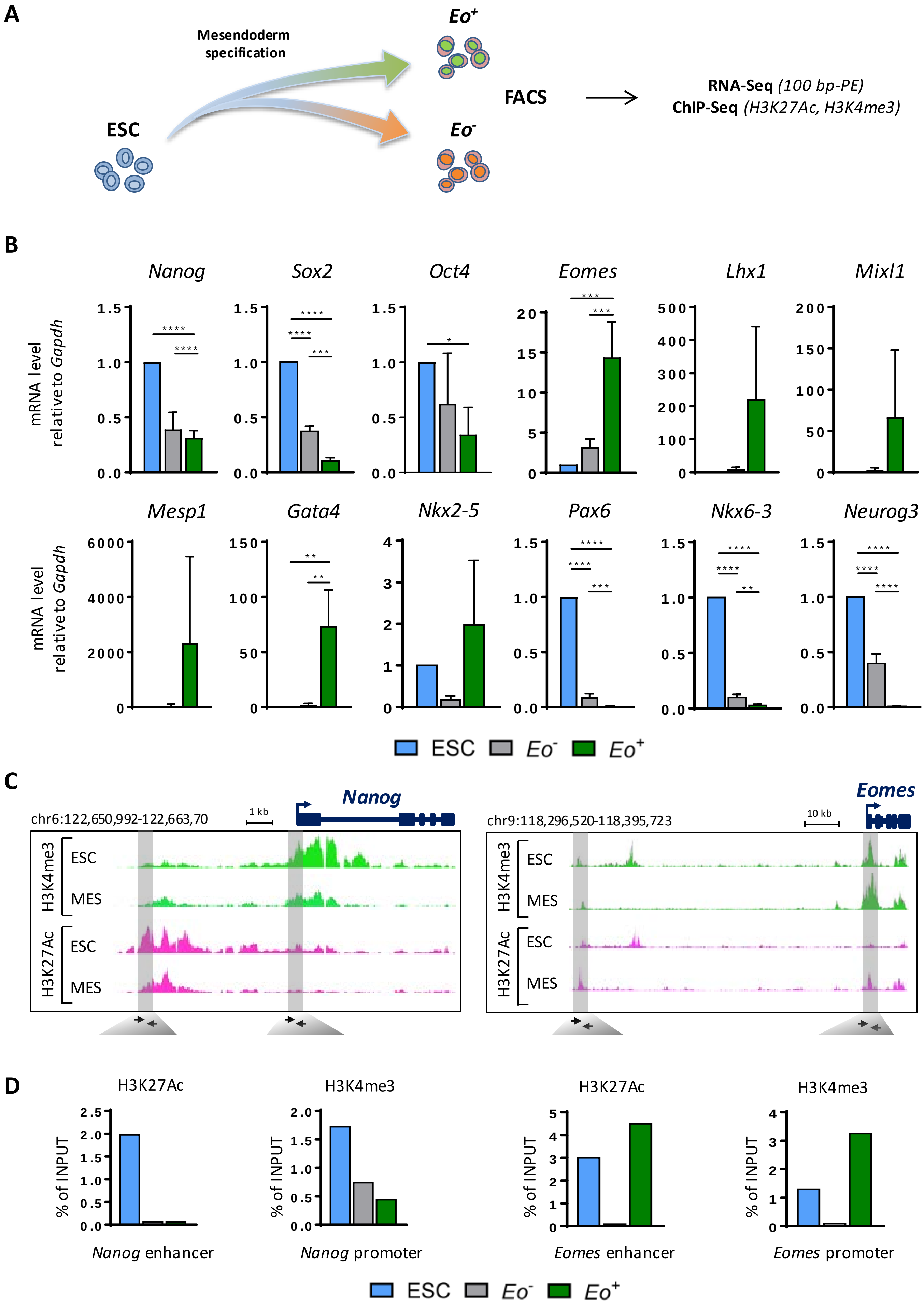


Suppl. Figure 2

**Supplementary Figure 2. Validation of the *Eomes* reporter mESC line** (**A**) RNAseq (100bp-PE) and ChIP-seq (H3K27Ac, H3K4me3) were performed on ESCs, and on differentiating cells at day3 sorted for *Eomes* expression (*Eomes* negative=$Eo^-$ and *Eomes* positive=$Eo^+$). (**B**) Markers of pluripotency, mesendoderm, cardiac mesoderm, early ectoderm and neuroectoderm in ESC, $Eo^-$ and $Eo^+$ cells quantified by qRT-PCR. Bars represent mean expression ± SEM (n=3) normalized to ESCs. *p* values were calculated using a one-way ANOVA test. (**C**) Schematic of the H3K4me3 (green) and H3K27Ac (pink) signal for the selected promoter and distal enhancer regions of *Nanog* and *Eomes* in ESCs and bulk mesodermal precursors. Selected promoters and distal enhancers are vertically highlighted (gray). The figure was created using data initially generated by Wamstad et al. (see reference [24]). (**D**) ChIP analysis: H3K27Ac and H3K4me3 enrichment at enhancers and promoters of *Nanog* and *Eomes.* Bars represent percentage of input. One representative biological replicate is shown. (* $P<0.05$; ** $P<0.01$; *** $P<0.001$; **** $P<0.0001$).

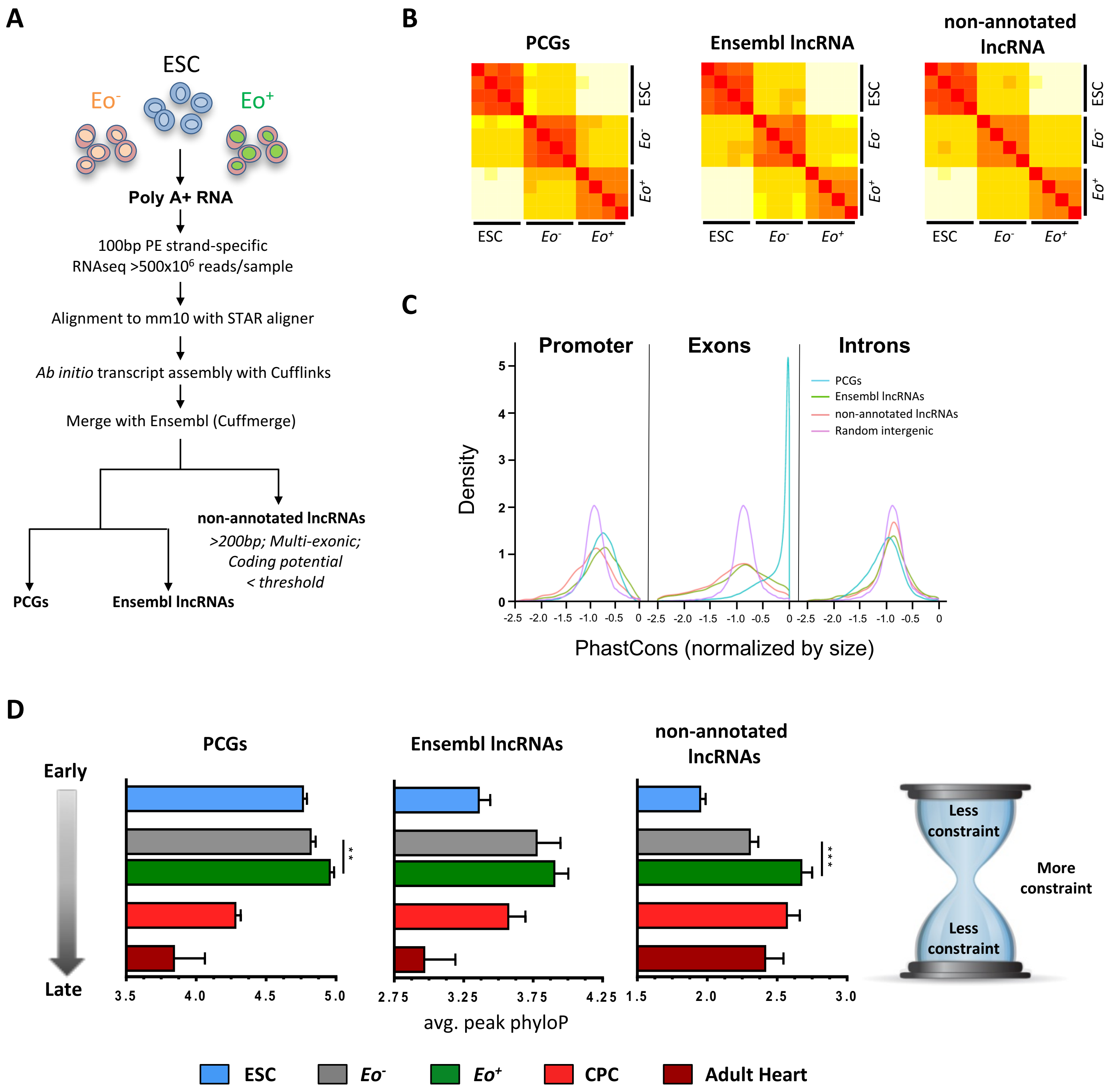


Suppl. Figure 3

**Supplementary Figure 3. Transcriptional and evolutionary characteristics of lncRNAs. Related to Figure 1.** (**A**) Schematic illustration of the procedure used to define PCGs, Ensembl lncRNAs and non-annotated lncRNAs. (**B**) Unsupervised clustering of PCGs, Ensembl lncRNAs, non-annotated lncRNAs in ESC, $Eo^-$ and $Eo^+$. (**C**) Kernel density plot of phastCons score distribution of PCGs, Ensembl lncRNAs, non-annotated lncRNAs and random intergenic sequence for promoters, exons and introns. (**D**) Bars show the mean evolutionary conservation (phyloP, x axis) at the proximal promoter DNA sequences of PCGs, Ensembl lncRNAs and non-annotated lncRNA enriched in ESCs, $Eo^-$, $Eo^+$, Cardiac Precursor Cells (CPCs) and in the adult heart. Statistical significance is just shown between $Eo^-$ and $Eo^+$. *p* values were calculated using a two-tailed t test. (** $P<0.01$; *** $P<0.001$).

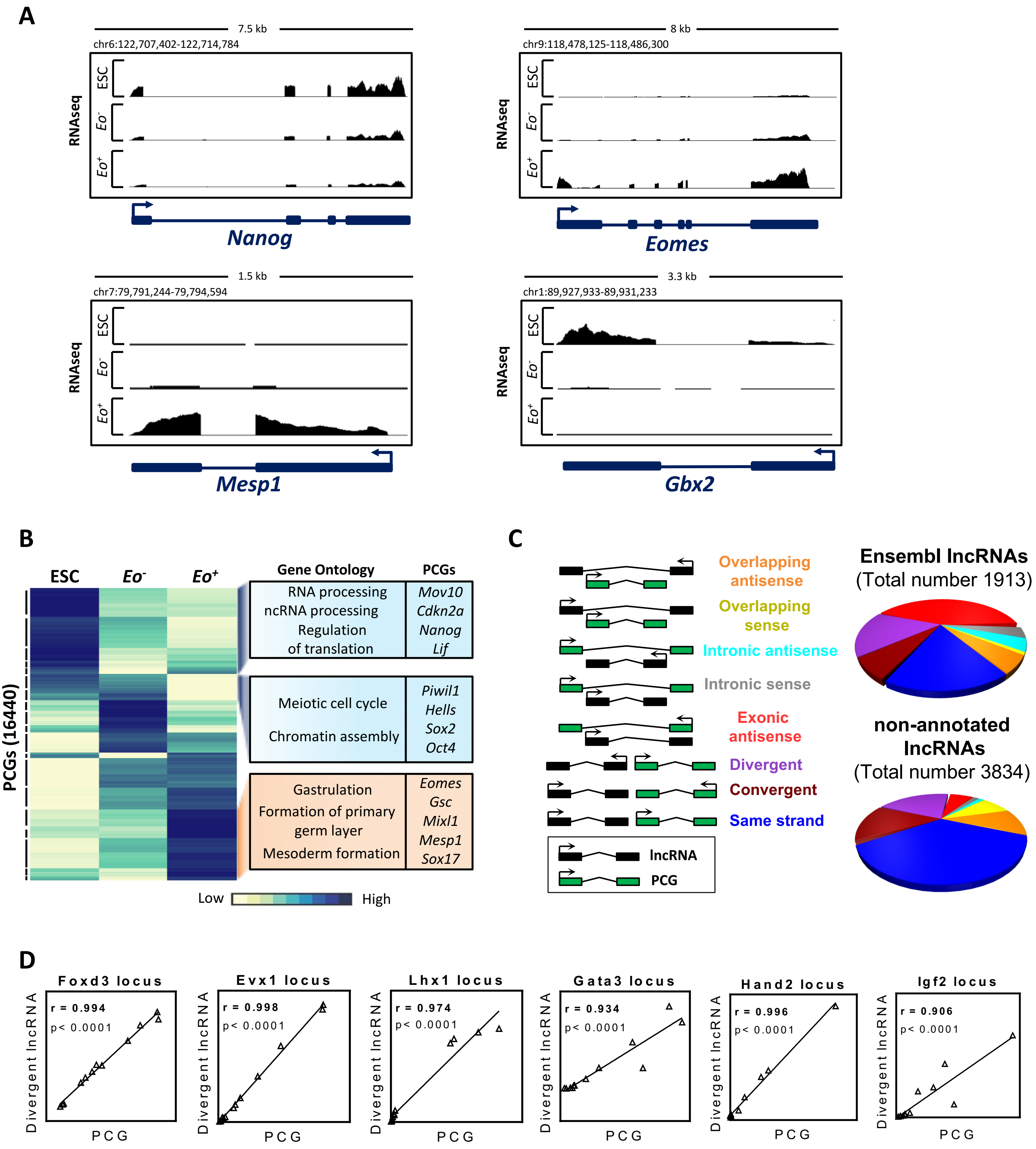


**Suppl. Figure 4**

**Supplementary Figure 4. Stage specific expression of PCGs and lncRNA biotypes. Related to Figure 1.** (**A**) RNAseq reads in ESC, *Eo*$^-$ and *Eo*$^+$ at the *Nanog*, *Eomes*, *Mesp1* and *Gbx2* loci. (**B**) Hierarchical clustering of PCG expression across ESC, *Eo*$^-$ and *Eo*$^+$. Enriched GO terms and examples PCGs are shown to the right. (**C**) Distribution of the different biotypes of Ensembl and non-annotated lncRNAs based on their genomic location and orientation as compared to the closest PCG. (**D**) Expression correlation between *Foxd3*, *Evx1*, *Lhx1*, *Gata3*, *Hand2* and *Igf2* and their cognate divergent lncRNAs. Pearson's correlation test (r; 95% CI).

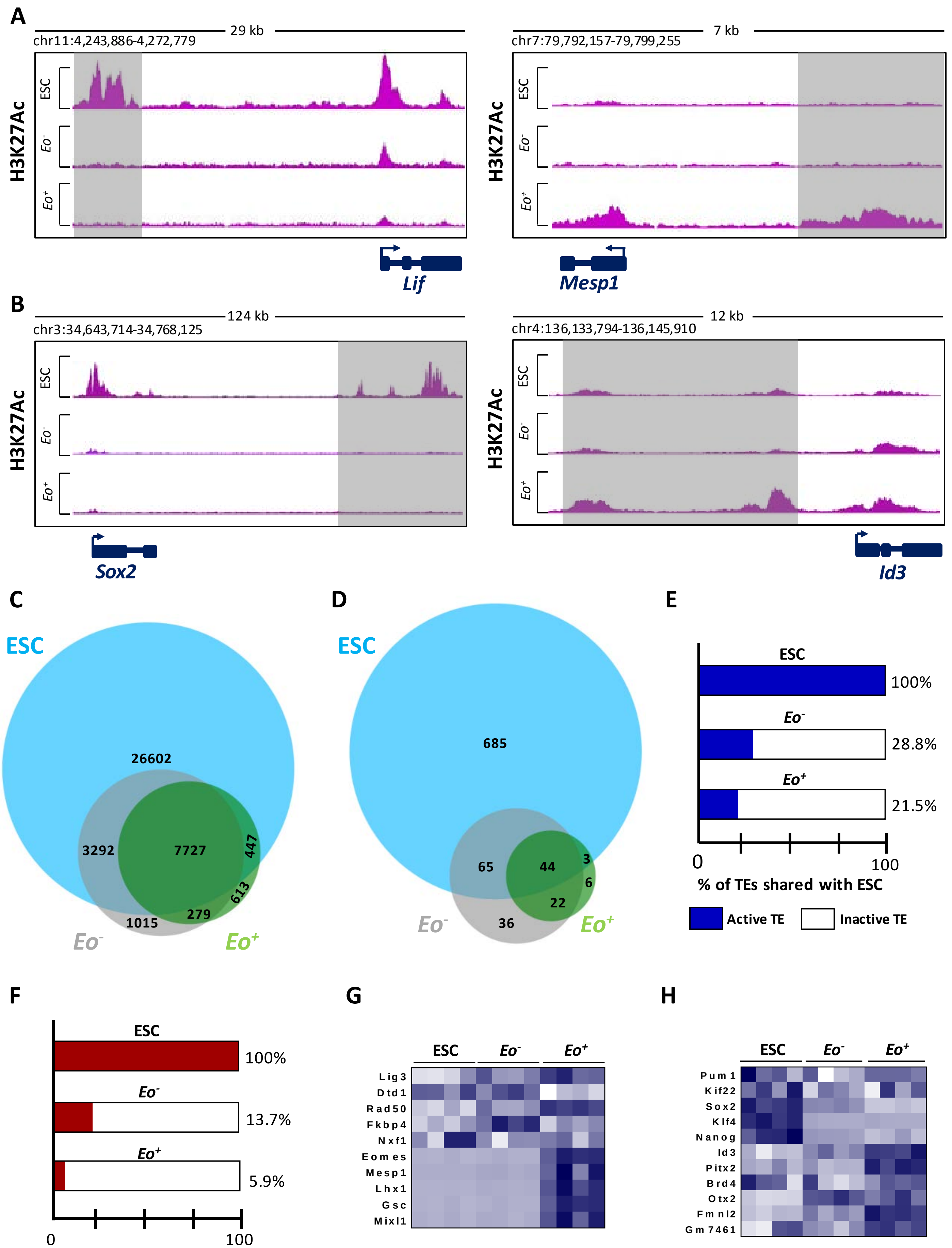


Suppl. Figure 5

**Supplementary Figure 5 Restriction of the enhancer landscape during mesendoderm specification. Related to Figure 3.** (**A**) Example of TE regions. H3K27Ac signal in ESCs, *Eo*$^-$ and *Eo*$^+$ at the *Lif1* and *Mesp1* loci. TE regions active in at least one stage of differentiation are vertically highlighted (gray). (**B**) Example of SE regions. H3K27Ac signal in ESCs, *Eo*$^-$ and *Eo*$^+$ at the *Sox2* and *Id3* loci. SE regions active in at least one stage of differentiation are vertically highlighted (gray). (**C**) Venn diagram depicts shared and unique TEs in ESC, *Eo*$^-$ and *Eo*$^+$. (**D**) Venn diagram depicts shared and unique SEs in ESC, *Eo*$^-$ and *Eo*$^+$. (**E**) Percentages of TEs shared with ESCs in *Eo*$^-$ and *Eo*$^+$. (**F**) Percentages of SEs shared with ESCs in *Eo*$^-$ and *Eo*$^+$. (**G**) Expression heatmap of representative PCGs shown in Figure 3D in ESC, *Eo*$^-$ and *Eo*$^+$. (**H**) Expression heatmap of representative PCGs shown in Figure 3E in ESC, *Eo*$^-$ and *Eo*$^+$.

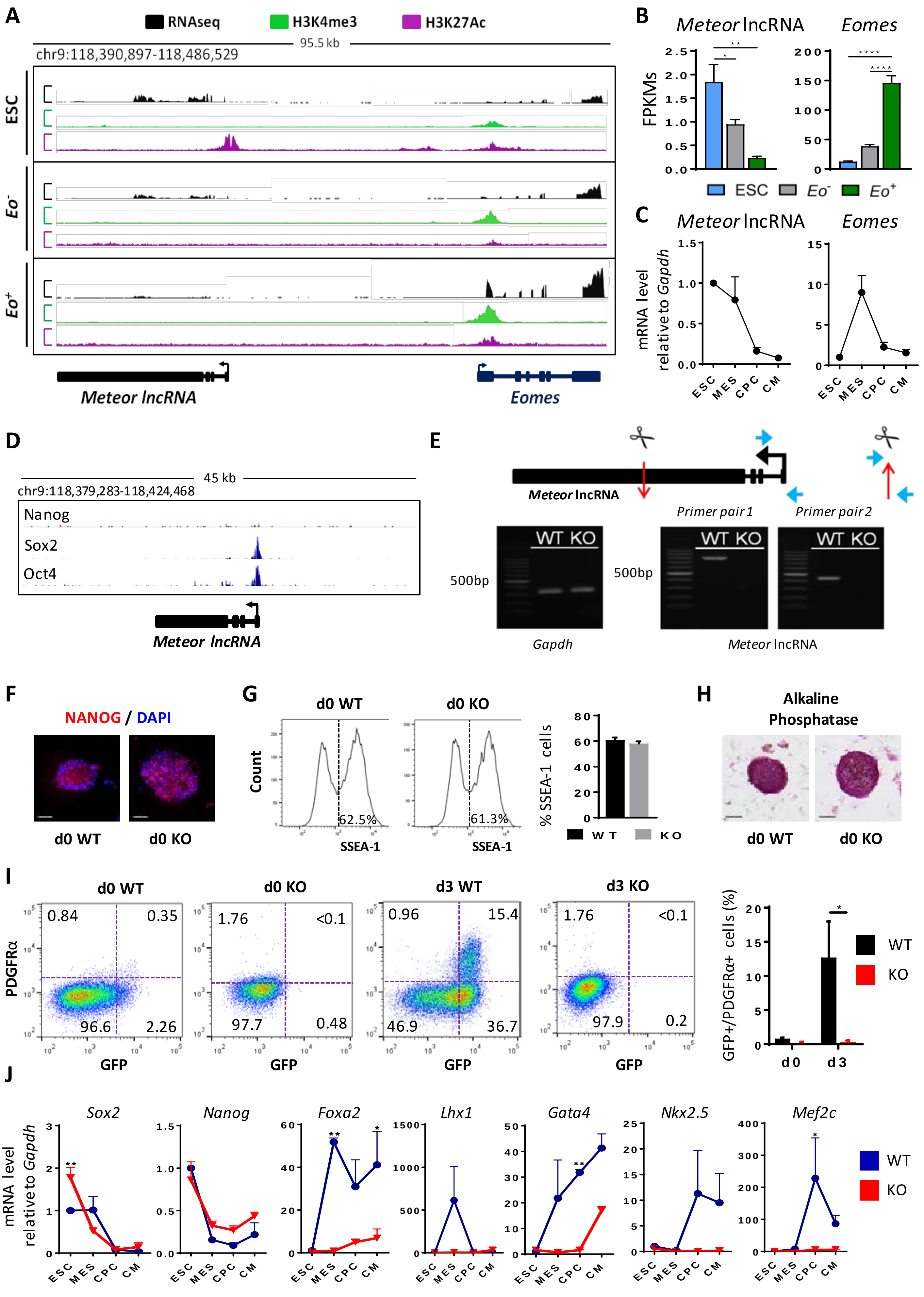


**Suppl. Figure 6**

**Supplementary Figure 6. *Meteor*-KO cells fail to give rise to ME-derived cells and maintain fundamental stemness features. Related to Figure 6.** (**A**) RNAseq, H3K4me3 and H3K27Ac reads in ESCs, $Eo^-$ and $Eo^+$ at the *Eomes* and *Meteor* loci. (**B**) RNAseq expression (FPKMs) of *Meteor* lncRNA and *Eomes* in ESC, $Eo^-$ and $Eo^+$. Bars represent mean expression ± SEM (n=4). *p* values were calculated using a one-way ANOVA test. (**C**) Expression kinetic of *Meteor* lncRNA and *Eomes* during cardiogenic differentiation measured by qRT-PCR. Trends represent average of fold change (n=3 biological replicates) normalized to WT ESC. (**D**) Nanog, Sox2 and Oct4 ChIP-seq data in mouse ESCs at the *Meteor* locus. (**E**) Genotyping in *Meteor* KO cells. Two pairs of primers were designed in different regions of the deletion. (**F**) Immunochemistry on day0 colonies in WT and *Meteor* KO (NANOG: red; DAPI: blue). Scale represents 50 µm. (**G**) FC analysis: SSEA-1 profile at day0 in WT and *Meteor* KO. Percentage of SSEA-$1^+$ cells at day0 is shown to the right. Bars represent average of SSEA-$1^+$ cells (n=3 biological replicates). (**H**) Alkaline phosphatase assay on day0 colonies in WT and *Meteor* KO. Scale bar represents 100 µm. (**I**) FC analysis: GFP and PDGFRα analysis on day0 and day3 in WT and *Meteor* KO. Percentage of $GFP^+$/PDGFR$\alpha^+$ cells is shown to the right. Bars represent $GFP^+$/PDGFR$\alpha^+$ cells in WT and *Meteor* KO at day0 and day3. *p* values were calculated using a two-tailed t test. (**J**) Expression kinetic of *Sox2*, *Nanog*, *Foxa2*, *Lhx1*, *Gata4*, *Nkx2-5* and *Mef2c* in WT and KO cells during cardiogenic differentiation measured by qRT-PCR. Trends represent average of fold change (n=3 biological replicates) normalized to WT ESC. *p* values were calculated using a two-way ANOVA test. (* $P<0.05$; ** $P<0.01$; **** $P<0.0001$).

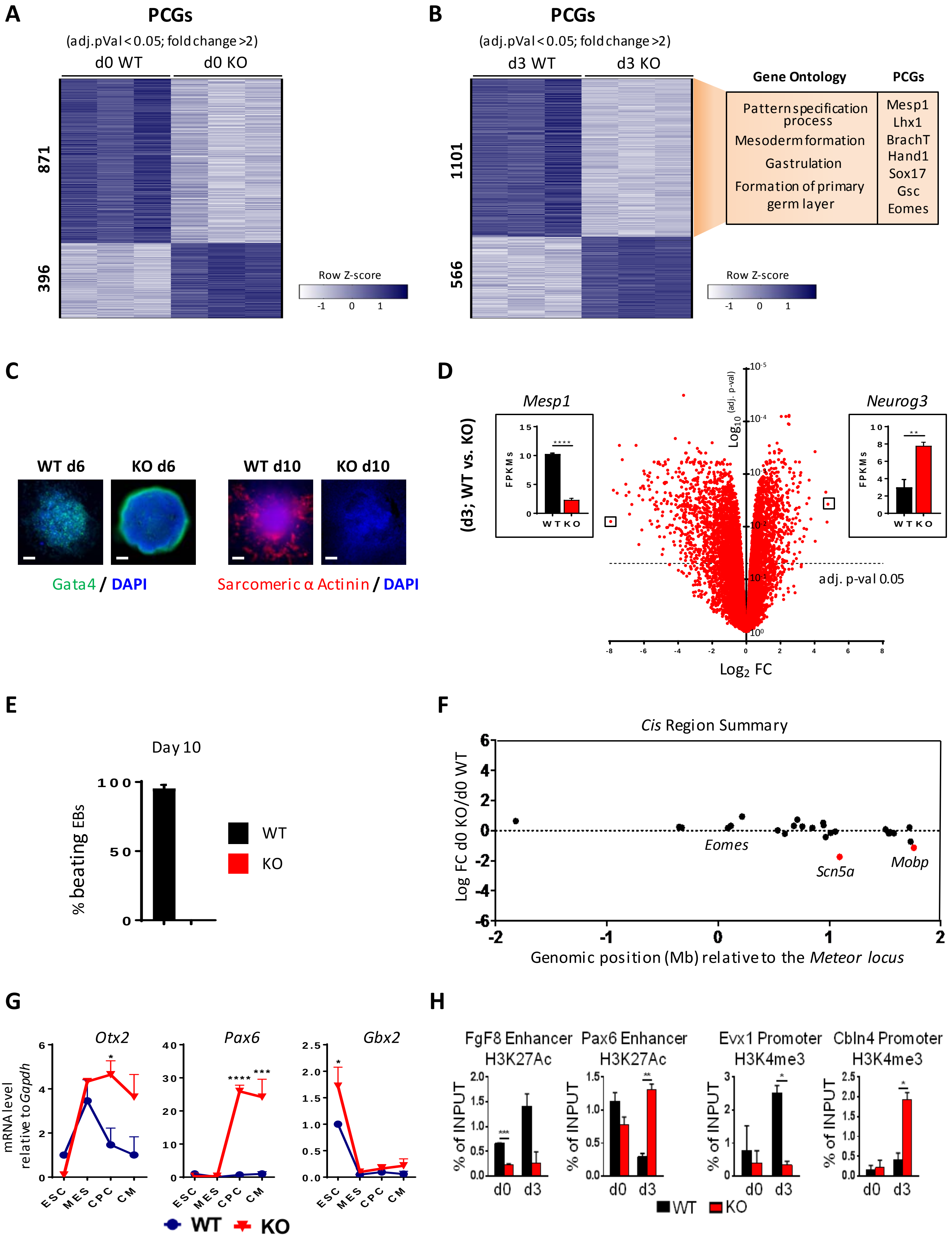


**Suppl. Figure 7**

**Supplementary Figure 7. *Meteor*-KO cells are transcriptionally and epigenetically redirected towards the neuroectoderm fate. Related to Figure 6.** (**A**) Hierarchical clustering of differentially expressed PCGs (FC>2) in d0 WT and d0 *Meteor* KO. (**B**) Hierarchical clustering of differentially expressed PCGs (FC>2) in d3 WT and *Meteor* KO d3. Enriched GO terms and example PCGs are shown to the right. (**C**) Anti-GATA4 and anti-Sarcomeric α ACTININ antibody staining on day6 and day10 WT and *Meteor* KO EBs. (GATA4: green; Sarcomeric α ACTININ:red; DAPI: blue). Scale bar represents 100 μm. (**D**) Volcano plot representation of expressed PCGs in d3 WT and d3 KO. Expression (RNA Seq data; FPKMs) of *Mesp1* and *Neurog3* in WT and *Meteor* KO at day3 is shown. Bars represent mean expression ± SEM (n=3). *p* values were calculated using a two-tailed t test. (**E**) Percentage of beating embryoid bodies at day10 in WT and *Meteor* KO. (**F**) Summary of local transcriptional changes upon *Meteor* deletion (±2 Mb of *Meteor* locus) in d0 WT and d0 KO. Dots represent LogFC of PCGs. Red indicates significant differential expression. (**G**) Expression kinetic of *Otx2*, *Pax6* and *Gbx2* in WT and *Meteor* KO cells during cardiogenic differentiation measured by qRT-PCR. Trends represent average of fold change (n=3 biological replicates) normalized to WT ESC. *p* values were calculated using a two-way ANOVA test. (**H**) ChIP analysis: H3K27Ac enrichment at distal enhancers of *FgF8* and *Pax6* and H3K3me3 enrichment at promoters of *Evx1* and *Cbln4* in d0 WT, d0 KO, d3 WT and d3 KO. Bars represent average of percentage of input (n=3 biological replicates). *p* values were calculated using a two-tailed t test. (* *P*<0.05; ** *P*<0.01; *** *P*<0.001;**** *P*<0.0001).

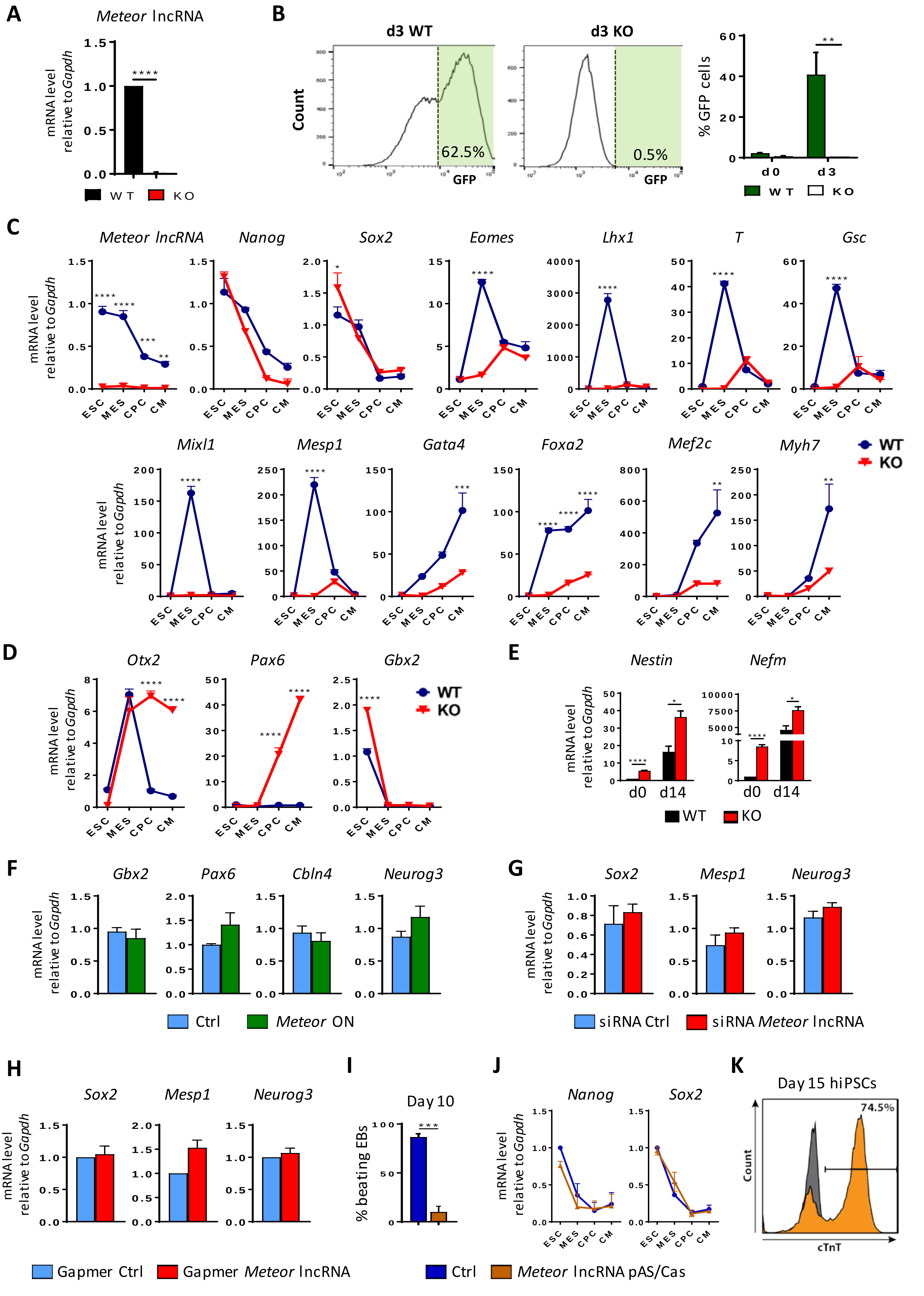


**Suppl. Figure 8**

**Supplementary Figure 8. Additional characterization of the *Meteor* locus. Related to Figure 6 and 7.** (**A**) *Meteor* lncRNA expression in WT and *Meteor* KO ESCs (additional clone) measured by qRT-PCR. Bars represent average of fold change (n=3 biological replicates) normalized to WT. *p* value was calculated using a two-tailed t test. (**B**) FC analysis: GFP profile at day0 and day3 of differentiation in WT and *Meteor* KO (additional clone). Percentage of $GFP^+$ cells at day0 and day3 in WT and *Meteor* KO are shown to the right. Bars represent average of GFP+ cells (n=3 biological replicates). *p* values were calculated using a two-tailed t test. (**C**) Expression kinetic of *Meteor lncRNA*, *Nanog*, *Sox2*, *Eomes*, *Lhx1*, *T*, *Gsc*, *Mixl1*, *Mesp1*, *Gata4*, *Foxa2*, *Mef2c* and *Myh7* in WT and *Meteor*-KO (additional clone) cells during cardiogenic differentiation measured by qRT-PCR. Trends represent average of fold change (n=3 biological replicates) normalized to WT ESC. *p* values were calculated using a two-way ANOVA test. (**D**) Kinetics of *Otx2*, *Pax6* and *Gbx2* expression in WT and *Meteor*-KO (additional clone) cells during cardiogenic differentiation measured by qRT-PCR. Trends represent average of fold change (n=3 biological replicates) normalized to WT ESC. *p* values were calculated using a two-way ANOVA test. (**E**) *Nestin* and *Nefm* expression in WT and *Meteor*-KO (additional clone) cells at d0 and d14 of differentiation measured by qRT-PCR. Bars represent average of fold change (n=3 biological replicates) normalized to day0 WT. *p* values were calculated using a two-tailed t test. (**F**) *Gbx2*, *Pax6*, *Cbln4* and *Neurog3* expression in P19CL6 cells following CRISPR-on-mediated *Meteor lncRNA* induction. Mean ± SEM (n=4) normalized to Ctrl. *p* values were calculated using a two-tailed t test. (**G**) *Sox2*, *Mesp1* and *Neurog3* expression in ESCs following transfection with siRNAs targeting *Meteor lncRNA* or a control sequence. Mean ± SEM (n=3) normalized to siRNA Ctrl. *p* values were calculated using a two-tailed t test. (**H**) *Sox2*, *Mesp1* and *Neurog3* expression in ESCs following transfection with GapmeRs targeting *Meteor lncRNA* or a control sequence. Mean ± SEM (n=3) normalized to Gapmer Ctrl. *p* values were calculated using a two-tailed t test. (**I**) Percentage of beating embryoid bodies at day10 in Ctrl and *Meteor lncRNA* pAS/Cas cells. *p* values were calculated using a two-tailed t test. (**J**) Expression kinetics of pluripotency genes in Ctrl and *Meteor lncRNA* pAS/Cas cells during cardiogenic differentiation measured by qRT-PCR. Trends represent average of fold change (n=3 biological replicates) normalized to Ctrl ESC. *p* values were calculated using a two-way ANOVA test. (**K**) FACS analysis: cTnT profile at day15 of human iPSC-derived cardiomyocytes showing high cell purity based on directed differentiation protocol. (* $P<0.05$; ** $P<0.01$; *** $P<0.001$; **** $P<0.0001$).

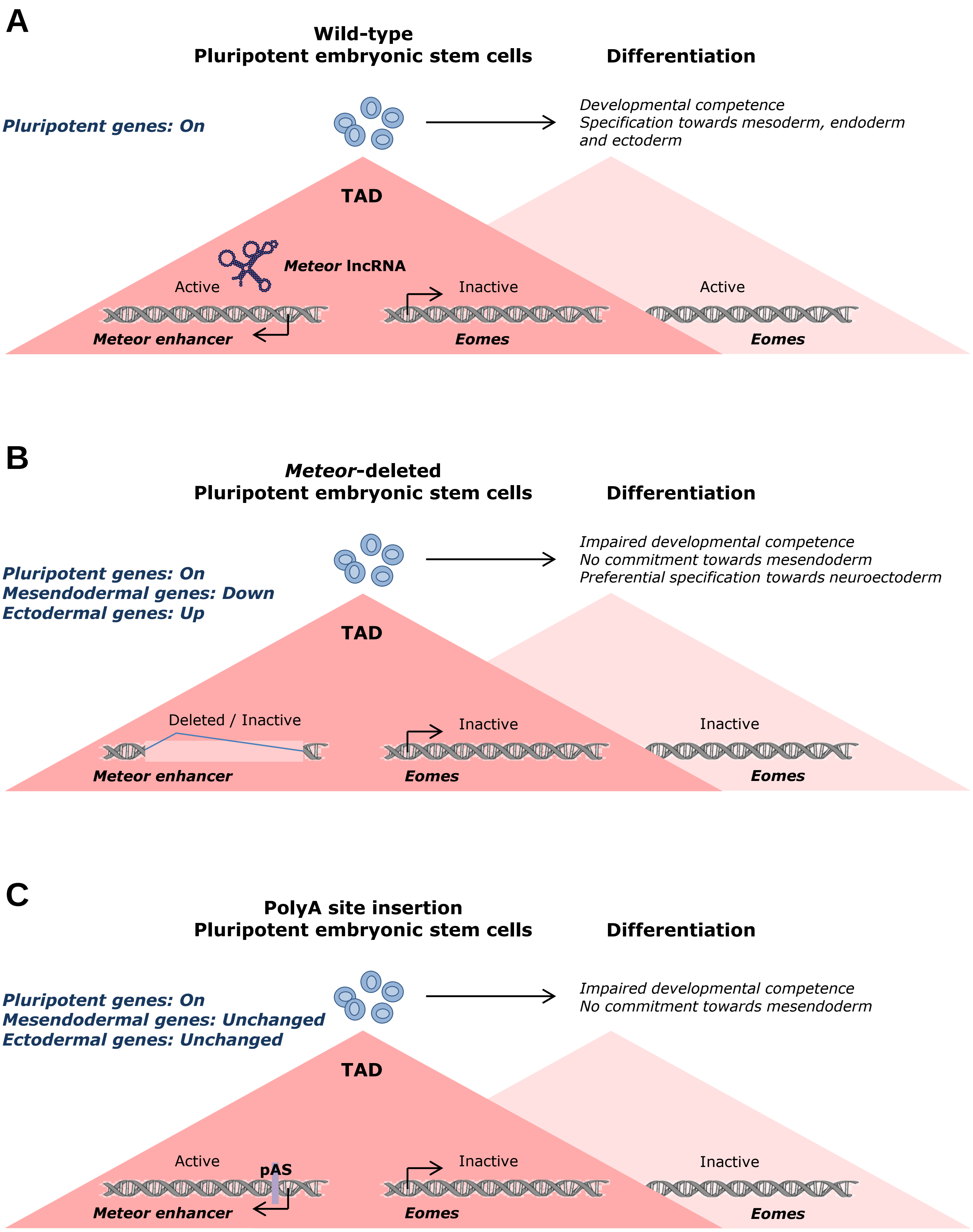


Suppl. Figure 9

**Supplementary Figure 9. The *Meteor* locus in pluripotency and differentiation.** (**A**) ESCs possess developmental competence for all the three germ layers. *Meteor* enhancer is active in pluripotency and coupled with high expression of *Meteor* lncRNA. When ESCs receive the inductive signal for specification the *Meteor* enhancer is shut off, *Eomes* is expressed and ESCs can differentiate towards ME and ectoderm. (**B**) When the *Meteor* enhancer is deleted ESCs lose their developmental competence for ME specification and are not able to differentiate into the ME lineage. In contrast, *Meteor* KO cells show increased commitment into the ectoderm/neuroectoderm fate in pluripotency and during differentiation. (**C**) When transcription at the *Meteor* locus is reduced and *Meteor* lncRNA expression abrogated ESCs maintain their developmental competence for ME specification. In contrast, when differentiated, they show impaired commitment towards the ME lineage.

**Supplementary Table 1**. List of Taqman probes and primers used in real-time qPCR analysis.

| Species | Gene name | Taqman probe reference | Supplier |
|---|---|---|---|
| Mouse | *Gapdh* | Mm99999915_g1 | Applied biosystem |
| Mouse | *Nanog* | Mm_02384862_g1 | Applied biosystem |
| Mouse | *Sox2* | Mm_00488369_s1 | Applied biosystem |
| Mouse | *Eomes* | Mm_01351985_m1 | Applied biosystem |
| Mouse | *T* | Mm_00436877_m1 | Applied biosystem |
| Mouse | *Mesp1* | Mm_00801883_g1 | Applied biosystem |
| Mouse | *Gata4* | Mm_00484689_m1 | Applied biosystem |
| Mouse | *Nkx2-5* | Mm_01309813_s1 | Applied biosystem |
| Mouse | *Mef2c* | Mm_01340842_m1 | Applied biosystem |
| Mouse | *Myh6* | Mm_00440354_m1 | Applied biosystem |
| Mouse | *Myh7* | Mm_00600555_m1 | Applied biosystem |
| Mouse | *Mixl1* | Mm_00489085_m1 | Applied biosystem |
| Mouse | Nestin | Mm_00450205_m1 | Applied biosystem |
| | | | |
| **Species** | **Gene name** | **Forward primer** | **Reverse primer** |
| Mouse | *Gapdh* | TGCACCACCAACTGCTTAGC | GGCATGGACTGTGGTCATGAG |
| Mouse | *Pax6* | TACCAGTGTCTACCAGCCAAT | TGCACGAGTATGAGGAGGTCT |
| Mouse | *Nkx6-3* | CGGTGCAGAACTCTTTCTACAA | TGGGAGCTGAGTCCACCAAA |
| Mouse | *Neurog3* | CCAAGAGCGAGTTGGCACT | CGGGCCATAGAAGCTGTGG |
| Mouse | *Meteor lncRNA* | CCCCTGTCAGCATAGCTTTT | TCGTGCTTGTCTCACTCCTG |
| Mouse | *Gsc* | CCCCGGTTCTGTACTGGTG | TCTGGGTACTTCGTCTCCTGG |
| Mouse | *Foxa2* | CCCTACGCCAACATGAACTCG | GTTCTGCCGGTAGAAAGGGA |
| Mouse | *Otx2* | TATCTAAAGCAACCGCCTTACG | AAGTCCATACCCGAAGTGGTC |
| Mouse | *Gbx2* | CAACTTCGACAAAGCCGAGG | ACTCGTCTTTCCCTTGCCCT |
| Mouse | *Nefm* | TCTTCGGCCTCTTCCTTCTC | CCAAGCTCAAGGTCCAACAC |

**Supplementary Table 2**. List of primers used in ChIP real time-PCR analysis.

| Species | Region name | Forward primer | Reverse primer |
|---|---|---|---|
| Mouse | *Nanog* Enhancer | GCCAACTGAACTACACGCTC | TGCCTCTGACTCTGGAAGAC |
| Mouse | *Nanog* Promoter | TCCAGCAGAAGCAGTTAGCA | GTTTGGAAGGAGCACTGAGC |
| Mouse | *Eomes* Enhancer | GATGAGAAACCGTCGTGTGG | GCCTGGACCCAACTTACTCA |
| Mouse | *Eomes* Promoter | TAGCGAAAGAGGGCTAGCTG | CTTACAGAAGAGCCCCACCA |
| Mouse | *Nkx6-3* Enhancer | CACCCAGCCAAGTCTCCTAA | TCCTTTGCTCCGTCTCTGT |
| Mouse | *Nkx6-3* Promoter | GAACAGCAGCCTCCTCTCTG | ACCTGGGGGCCATAGTAGAC |
| Mouse | *Lhx1* Enhancer | CAACCTTGACGCGAAAGACA | CCCAAACCTGTGCCCATATC |
| Mouse | *Lhx1* Promoter | AGGGCTCTGTAGTTCCGACA | GCTCTCCAGCAACCAAAGAC |
| Mouse | *Neurog3* Enhancer | GGCCAAGATAATGGAAAGCA | GCCACCATAAACTCCAGCAT |
| Mouse | *Neurog3* Promoter | TCCTCGGAGCTTTTCTACGA | GAGCAGCAGGTCAGTCAGTG |
| Mouse | *Fgf8* Enhancer | CATCTGGAGAGAGCGAGGTC | CTTCTCCCACAGCTCCTGTC |
| Mouse | *Pax6* Enhancer | CCTCTTCTGTCCAGGCTTTG | GCCGAGAGAGCTAGGGAACT |
| Mouse | *Evx1* Promoter | CGAGTACCAGCACAGCAAAG | CAAGGGAAAGGAGCATCTTG |
| Mouse | *Cbln4* Promoter | CGCCAAAAGCAGTTCTAAGG | TGCAGGCAGCACTCCTATAA |

**Supplementary Table 3**. List of gRNA used for generating Meteor KO.

| Species | gRNA name | gRNA sequence |
|---|---|---|
| Mouse | Meteor upstream gRNA 1 | GCTTCCCATTTGCGGTGTTGT |
| Mouse | Meteor upstream gRNA 2 | GAGGTTCTATGAGGTAGATAC |
| Mouse | Meteor downstream gRNA 1 | GCGAGTTCTATGGAACTCGGA |
| Mouse | Meteor downstream gRNA 2 | GCCGTATTTAAAGCAGGCCT |

**Supplementary Table 4**. List of primers used to genotype Meteor KO.

| Species | Primer name | Forward primer | Reverse primer |
|---|---|---|---|
| Mouse | Primer pair 1 | TTGGGTCTTGCAGCCTAGTT | CTGCCATCATCGTCATCATC |
| Mouse | Primer pair 2 | TATCACTTGCCATGCTCTGC | GAGATAGCCTTCCCCCAATC |

**Supplementary Table 5**. List of primers used in human iPSCs Cardiac-Directed Differentiation.

| Species | Gene name | Forward primer | Reverse primer |
|---|---|---|---|
| Human | *HPRT* | TGACACTGGCAAAACAATGCA | GGTCCTTTTCACCAGCAAGCT |
| Human | *Meteor lncRNA* | CCCAGCTGCATTGTTTACCT | GTCCTATGCCCACGGAGTCT |
| Human | *NANOG* | CATGAGTGTGGATCCAGCTTG | CCTGAATAAGCAGATCCATGG |
| Human | *T* | CAAATCCTCATCCTCAGTTTG | GTCAGAATAGGTTGGAGAATTG |
| Human | *EOMES* | CACATTGTAGTGGGCAGTGG | CGCCACCAAACTGAGATGAT |
| Human | *ISL1* | ATTTCCCTATGTGTTGGTTGC | CGTTCTTGCTGAAGCCGATG |
| Human | *TMEM88* | GCTGCCTTCAATCTTCTCCTG | ATAAAGGGCTCGGCTGTAGG |
