# Supplementary material for: A transcribed enhancer dictates mesendoderm specification in pluripotency
Source: Nat Commun. 2017 Nov 27;8:1806. doi: 10.1038/s41467-017-01804-w (PMC5703900; doi:10.1038/s41467-017-01804-w)
Supplement: Supplementary file 2 — Description of Additional Supplementary Files [file 41467_2017_1804_MOESM2_ESM.pdf]

Description of Additional Supplementary Files

File Name: Supplementary Data 1
Description: Unfiltered list of transcript expression in ESCs, Eo^- cells and Eo^+ cells.

File Name: Supplementary Data 2
Description: Gene Ontology terms related to PCGs in ESCs, Eo^- cells and Eo^+ cells.

File Name: Supplementary Data 3
Description: Gene Ontology terms related to PCGs adjacent to lncRNAs (Ensembl and Nonannotated) in ESCs, Eo^- cells and Eo^+ cells.

File Name: Supplementary Data 4
Description: Gene Ontology terms related to PCGs adjacent to TEs and SEs in ESCs, Eo^- cells and Eo^+ cells.

File Name: Supplementary Data 5
Description: Gene Ontology terms related to PCGs adjacent to plncRNAs, TE lncRNAs and SE lncRNAs in ESCs, Eo^- cells and Eo^+ cells.

File Name: Supplementary Data 6
Description: Unfiltered list of transcript expression in WT and *Meteor* KO ESCs.

File Name: Supplementary Data 7
Description: Gene Ontology terms related to PCGs in WT and *Meteor* KO ESCs.
